# Reduced butyrate-producing bacteria and altered metabolic pathways in the gut microbiome of immunoglobulin A nephropathy patients

**DOI:** 10.1038/s41598-025-13629-5

**Published:** 2025-07-31

**Authors:** Anna Popova, Kārlis Rācenis, Monta Brīvība, Rihards Saksis, Mikus Saulīte, Baiba Šlisere, Egija Berga-Švītiņa, Kristīne Oļeiņika, Anna Jana Saulīte, Jānis Seilis, Juta Kroiča, Harijs Čerņevskis, Aivars Pētersons, Jānis Kloviņš, Aivars Lejnieks, Viktorija Kuzema

**Affiliations:** 1 Department of Internal Diseases, Riga Stradins University, Riga, Latvia; 2 Center of Nephrology, Pauls Stradins Clinical University Hospital, Riga, Latvia; 3 Department of Biology and Microbiology, Riga Stradins University, Riga, Latvia; 4 Latvian Biomedical Research and Study Centre, Riga, Latvia; 5 Joint Laboratory, Pauls Stradins Clinical University Hospital, Riga, Latvia; 6 Institute of Oncology, Riga Stradins University, Riga, Latvia; 7 Program in Cellular and Molecular Medicine, Boston Children’s Hospital, Harvard Medical School, Boston, MA USA; 8 Department of Internal Medicine, University of Latvia, Riga, Latvia; 9 Riga East University Hospital, Riga, Latvia

**Keywords:** Gut microbiome, Immunoglobulin a nephropathy, Progressors, Butyrate-producing bacteria, IgA nephropathy, Microbiome

## Abstract

Gut-associated lymphoid tissue is central to the production of galactose-deficient IgA1 (Gd-IgA1), a key factor in immunoglobulin A nephropathy (IgAN). Although no major differences in gut microbiome diversity have been reported across IgAN cohorts, functional alterations in microbial composition may contribute to disease pathogenesis. The study was designed as a cross-sectional study with an embedded prospective cohort component. Forty-eight adults with biopsy-confirmed IgAN—categorized as progressors (eGFR decline > 5 ml/min/1.73 m²/year, n = 23) or nonprogressors (n = 23)—and 23 healthy controls (HC) were recruited. Stool samples underwent metagenomic and functional profiling. Alpha diversity did not differ significantly between IgAN patients and HC. However, butyrate-producing bacteria (Butyrococcus, Agathobacter rectalis) were less abundant in IgAN patients. The sulfoquinovose degradation I pathway, associated with these bacteria, was also reduced. Nucleotide- and nucleoside-biosynthesis pathways were elevated in IgAN. Gd-IgA1 levels correlated with variations in metabolic pathways. Progressors demonstrated enhanced activity in isopropanol biosynthesis, biotin biosynthesis II, and phospholipid biosynthesis pathways. IgAN patients show reduced butyrate-producing bacteria and distinct functional changes in the gut microbiome suggestive of immune activation and inflammation. Progressors exhibit additional metabolic shifts linked to bacterial membrane stabilization.

**Supplementary Information:**

The online version contains supplementary material available at 10.1038/s41598-025-13629-5.

## Introduction

Immunoglobulin A nephropathy (IgAN) is the most prevalent primary glomerular disease globally, with significant differences in its prevalence and presentation across various populations^1^. IgAN is more common in Asian populations than in Caucasians^2^. Across European countries, the estimated IgAN point prevalence is 2.53 per 10,000, ranging from 1.14 per 10,000 in Spain to 5.98 per 10,000 in Lithuania^3^. According to kidney biopsy registry data in Latvia, the incidence of IgAN is 13.2 cases per million people per year. Current evidence indicates that IgAN is not caused by a single pathogenic factor but is instead the result of multiple sequential pathogenic “hits”. Elevated levels of circulating polymeric galactose-deficient IgA1 (Gd-IgA1) and the production of O-glycan-specific antibodies contribute to the formation of IgA1-containing immune complexes. These complexes subsequently deposit in the mesangium, leading to inflammation and glomerular injury^4^. The geographic variation in the prevalence and heritable nature of Gd-IgA1 highlights the potential roles of both environmental factors and genetics in susceptibility to IgAN^5^. Although the exact site of Gd-IgA1 production remains to be determined, considerable evidence suggests that mucosa-associated lymphoid tissue is responsible for Gd-IgA1 production^6^. Genetic, microbial, and dietary factors, which interact to cause functional changes in the intestinal mucosal immune system, promote the development of IgAN^7^. Most of the studies that focused on gut microbiome characteristics in IgAN patients were performed in Asia^8^ with several conducted in Europe. The gut microbiome composition and microbial diversity vary in patients with IgAN compared with non-IgAN individuals and among progressors and nonprogressors^9^; however, their effects on the course of the disease are not fully understood.

## Methods

The study was designed as a cross-sectional study with an embedded prospective cohort component. Forty-eight adults with histologically proven IgAN without kidney replacement therapy selected from the kidney biopsy database at Pauls Stradins Clinical University Hospital, Latvia, were enrolled in the study from January 2020 until December 2022. This is the only facility diagnosing adult IgAN in Latvia and performing 85% of the country’s kidney biopsies; we adjusted population counts by 15% to ensure our database accurately represents the national IgAN patient group. Age- and sex-matched healthy volunteers were also enrolled. The twenty-three healthy controls had age-appropriate kidney function, with no active urine sediment and no presence of proteinuria. The exclusion criteria included pregnancy, diabetes mellitus, severe organ dysfunction, acute cardiovascular disease, hepatic diseases, acute or chronic autoimmune or infectious diseases, immunodeficiency, malignancies, alcohol abuse, and the use of antibiotic treatment in the last three months. All participants provided written informed consent.

Demographic and clinical information was recorded, physical examinations were performed, and peripheral blood, urine, and fecal samples were collected at baseline. Examinations were carried out in accordance with the relevant regulations. Serum levels of creatinine, urea, uric acid, albumin, and total cholesterol were measured using standard laboratory methods on the Atellica CH system (Siemens Healthineers, Erlangen, Germany). The glomerular filtration rate (eGFR) was calculated according to the CKD-EPI Creatinine Eq. (2021). The Gd-IgA1 level in the serum was measured using ELISA kit (Gd-IgA1 Assay Kit-IBL 30111694, IBL International GmBH, Germany) following the manufacturer’s instructions. The samples were diluted 200-fold with the provided EIA buffer, to obtain biomarker levels within the measurement range of the kit (1.56–100 ng/ml). Serum lipopolysaccharide (LPS) levels were detected by ELISA (MyBioSource MBS702450, San Diego, CA, USA). Proteinuria was measured using the spot protein-to-creatinine ratio (UPCR). Protein assessment in urine was performed on a Cobas Integra 400 plus (Roche Diagnostics GmbH, Mannheim, Germany). The red blood cell count in the urine was determined using the Atellica 1500 automated urinalysis system (Siemens Healthineers, Erlangen, Germany).

Pathohistological examination of kidney samples was performed using light microscopy, immunofluorescence, and electron microscopy. Kidney biopsy results were analysed according to the revised Oxford classification (MEST-C score).

**Fig. 1:**
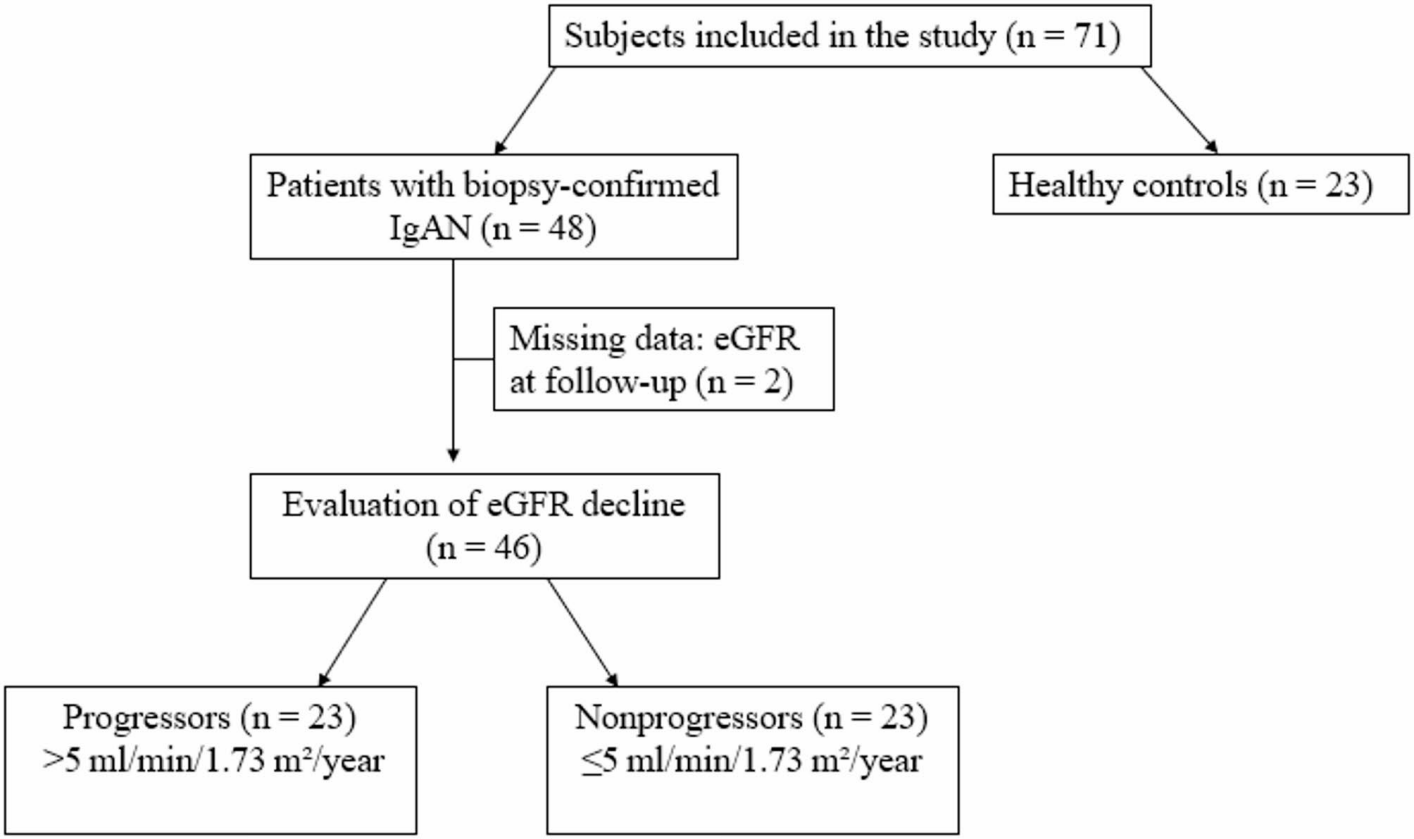
Study group flow chart.

Medical records were reviewed up to March 2024 to calculate the decrease in eGFR during the follow-up period. Progressors were defined as patients with an eGFR decline of more than 5 ml/min/1.73 m^2^/year (*n* = 23), whereas nonprogressors had an eGFR slope of 5 ml/min/1.73 m^2^/year or less (*n* = 23) (Fig. 1).

### Stool sample collection, microbial DNA isolation and shot-gun sequencing of DNA libraries

Stool samples were collected in two aliquots using sterile, buffer-free collection tubes. Each participant documented the precise date and time of sample collection. The samples were then aliquoted into cryogenic storage vials and stored at − 80 °C until analysis. The extraction of microbial DNA was carried out using the MGISP-960 Automated Sample Preparation System (MGI Tech Co., Ltd., Wuhan, China) and the MagPure Stool DNA LQ Kit (Angen Biotech Co., Ltd., Guangzhou, China). DNA library preparation was performed using the MGIEasy Universal DNA Library Prep Set (MGI Tech Co., Ltd., Wuhan, China) following the manufacturer’s instructions. The subsequent sequencing was conducted on the DNBSEQ-G400RS platform using the DNBSEQ-G400RS high-throughput sequencing set (PE 150) (MGI Tech Co., Ltd., Wuhan, China), which provides at least 20 million 150 bp paired-end sequencing reads per sample. The quantity and quality of the DNA were assessed using a Qubit 2.0 fluorometer (Thermo Fisher Scientific, Waltham, MA, USA) and an Agilent 2100 Bioanalyzer system (Agilent Technologies, Santa Clara, CA, USA), respectively.

### Processing and data analysis

The obtained clinical, anthropometric and biochemical data were processed using IBM SPSS Statistics 26. Parameters are reported as median values with interquartile ranges, and frequencies are expressed as percentages. For metagenomic shotgun sequencing data read quality evaluation and trimming was done using fastp v0.20.0^10^ with default parameters and by retaining only those reads that passed the 100 bp length requirements after the trimming. Host read contamination was controlled by mapping the reads against a *Homo sapiens* GRCh38 reference genome with Bowtie2 v2.3.5.1^11^ and removing the mapped reads. Taxonomic classification was performed by using Kracken2 v2.0.8^12^ with a confidence parameter set at 0.1 in accordance with the Unified Human Gastrointestinal Genome (UHGG)^13^ collection. Functional profiling was carried out using the HUMAnN 3.0^14^ software.

Statistical analysis for the phylum, genus and species taxonomic levels was performed by using the phyloseq v1.48.0^15^ microbiome analysis environment. First, we performed frequency and prevalence filtering to exclude taxa that had no counts in any of the samples and samples that had no counts in any of the detected taxa. Alpha diversity indices were calculated using the microbiome v1.26.0^16^ package, which was subsequently used as an input for the Wilcoxon rank sum test in the ggpubr v0.6.0^17^ package with Benjamini‒Hochberg p-value correction. Sample and group-level taxon proportion plots were produced from proportionally transformed counts using the MicrobiotaProcess v1.16.1^18^ package. Aitchison’s distance was calculated and plotted as a principal component analysis (PCA) plot for taxa passing the 10% sample prevalence threshold using the MicroViz v0.12.5^19^ package to represent the beta diversity results. Significant covariate detection was performed via the canonical correspondence analysis (CCA) ordinance method and PERMANOVA test within the MicroViz package, with 99,999 permutations. The resulting significant covariates were included in the MaAsLin2 v1.18.0^20^ design formula for the negative binomial analysis method of differentially abundant taxa detection with trimmed mean normalization applied beforehand, while the default values of the remaining parameters were used, except for increasing the taxon prevalence threshold from 10 to 33% in the case‒control species level test, to reduce the number of sporadic results. The same analysis methodology was applied to investigate the functional alterations in our experimental group. The default false discovery rate (FDR) threshold used in MaAsLin2 and also other microbiome studies is < 0.25. This relatively permissive threshold reflects the high dimensionality and inherent variability of microbiome data, where stringent cutoffs may overlook biologically relevant associations. Taxon-level associations with FDR-adjusted q-values below 0.25 are therefore commonly interpreted as statistically significant, while still requiring independent validation to confirm their biological relevance^21–23^. Visualizations were created using ggplot2 v3.5.1^24^ or the previously specified packages.

## Results

This study recruited forty-eight patients with IgAN and twenty-three healthy controls. The baseline and clinical characteristics are summarized in Table 1. The study participants were sex- and age-matched, with more than 60% being men, and ages ranging from 22 to 65 years. Both groups had a similar median body mass index (BMI) of 26 kg/m². As expected, serum creatinine levels were higher in patients with IgAN than in healthy controls. The distribution of IgAN patients across chronic kidney disease (CKD) stages was approximately 20%, with the majority in the CKD stage 1 group (33.3%). According to the pathological Oxford classification, approximately 70% of IgAN patients had mesangial hypercellularity (M1) and segmental glomerulosclerosis (S1) findings in their biopsy results. Patients had mild proteinuria, with a median UPCR of 0.349 g/g. Gd-IgA1 levels were greater in IgAN patients than in healthy individuals.

**Table 1:** Clinical, laboratory and pathological characteristics of patients with IgA nephropathy and healthy controls.

Baseline characteristics	IgAN patients, *n* = 48	HC, *n* = 23	*p*-value
Age, yr	41 (35–47.7)	46 (IQR 33–53)	0.36
Male	30 (62.5%)	14 (60.9%)	0.89
BMI, kg/m^2^	26.1 (23.6–29.3)	26 (23–28)	0.93
Systolic BP, mmHg	130 (121–145)	127 (120–138)	0.2
Diastolic BP, mmHg	80 (72.2–90)	80 (70–80)	0.3
Serum creatinine, µmol/l	105 (84–195.5)	80 (66–88)	0.001
eGFR, ml/min per 1.73m^2^	72.5 (32–97.7)	101 (96–108)	0.001
Gd-IgA1, ng/ml	6568 (4916–9335.5)	4577.5 (3470.3–6292.8)	0.02
Lipopolysaccharides, pg/ml	132.7 (107.2–189.5)	115 (89.9–230)	0.15
Indoxyl sulfate, ng/ml	240.7 (195.5–373.6)	230 (185–291)	0.69

The results are expressed as the median (interquartile range) or n (%).

### IgAN-specific gut microbiome signatures

**Fig. 2:**
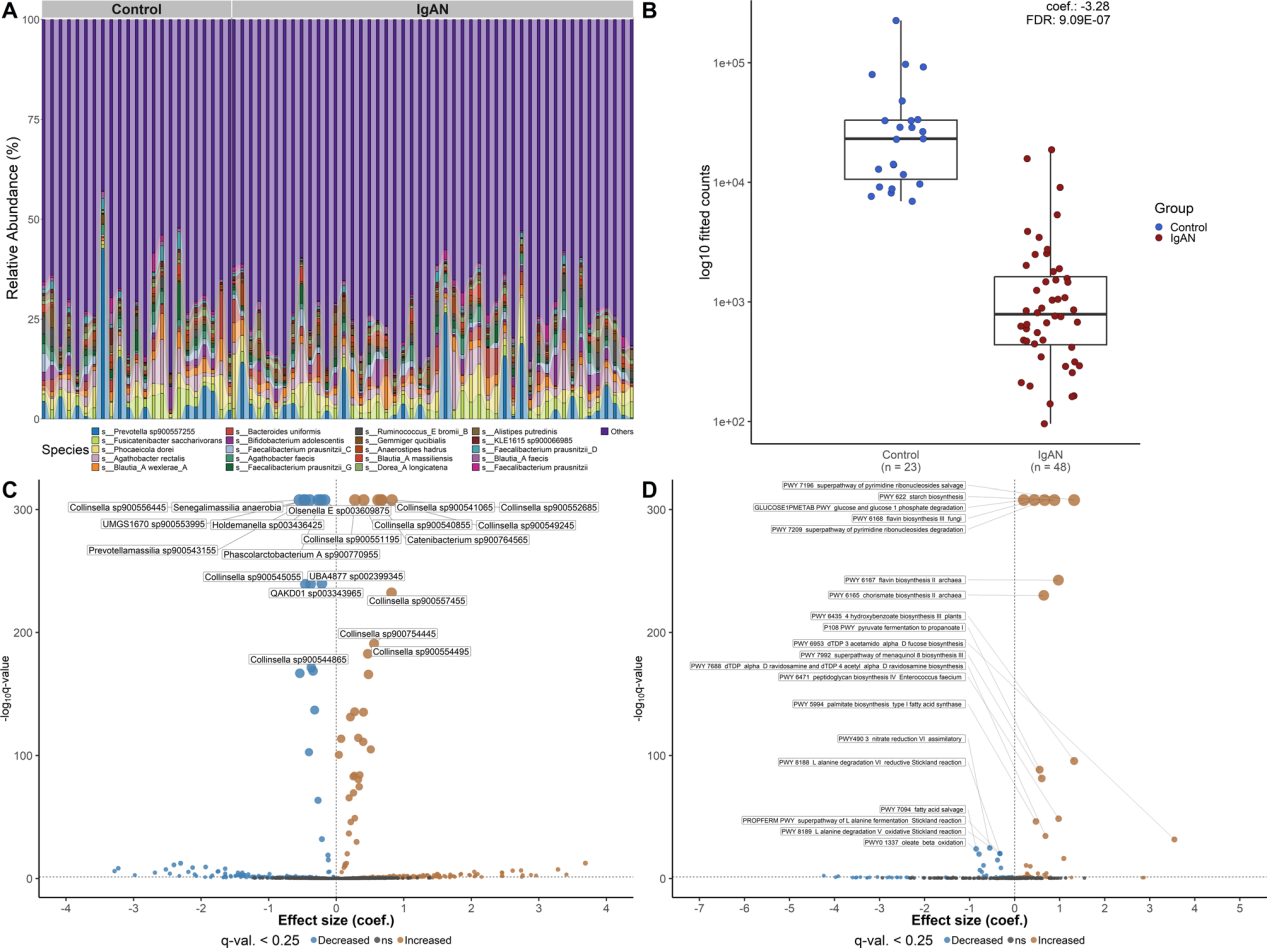
Alterations in the gut microbiome in IgA nephropathy patients and healthy individuals. **A** Taxonomy bar plot depicting 20 of the most representative species of every individual stratified by study group. **B** Relative abundance of *Eubacterium R sp000433975* in control subjects (blue) and IgAN patients (red). Boxplots show the median, 25th, and 75th percentiles and outliers. **C** Volcano plot representing the differential abundance effect size (logFC) and FDR relationship between control subjects and IgAN patients. Labeled values represent the top 20 Species based on FDR. A positive coefficient (brown) represents taxa with increased abundance in the IgAN patients, and a negative coefficient (blue) represents taxa in the control patients. Model adjusted for BMI. **D** Volcano plot of MetaCyc metabolic differentially abundant pathways. Labeled values represent the top 20 metabolic pathways based on FDR. The effect size is expressed as a coefficient with positive values for pathways enriched in IgAN patients, while negative values correspond to pathways enriched in control subjects. Model adjusted for Gd-IgA1, eGFR and BMI. **C** and **D** FDR values of 0 were replaced with the smallest non-zero normalized floating-point number in the R statistical programming language (2.225074e-308), to allow for logarithmic transformation and visualization. Covariates included in the models were selected based on association with microbiome variation as determined by PERMANOVA analysis.

To evaluate the IgAN-related alterations in the gut microbiome profiles, we compared the shotgun sequencing-derived gut microbiome profiles of fecal samples from IgAN patients (*n* = 48) with those of samples collected from healthy controls (*n* = 23). We observed no statistically significant difference in alpha diversities estimated by several common indices (Supplementary Table 1, Supplementary Fig. 1) between interest groups. In addition, no significant difference in the intersample variability in the microbial community composition among the analysed samples between the case and control groups was observed (Supplementary Table 2, Supplementary Fig. 2). *Prevotella* was the most prevalent genus in both IgAN patients (7.57%) and controls (9.27%), with *Faecalibacterium* (IgAN = 6.71%, controls = 8.11%) and *Blautia* (IgAN = 6.73%, controls = 7.02%) also among the most common genera. Notably, UBA4372 (coefficient = 6.74; FDR = 2.85E-04), a member of the Bacteroidaceae family, and RUG572 (coefficient = 6.66; FDR = 6.62E-16), belonging to the UBA1067 family within the Verrucomicrobiota phylum, were more abundant in IgAN patients than in controls. *Prevotella sp00900557255* (IgAN = 2.95%, controls = 4.34%), *Fusicatenibacter saccharivorans* (IgAN = 2.53%, controls = 2.58%), and *Phocaeicola dorei* (IgAN = 2.51% and controls = 2.28%) were the three most abundant species in both groups (Supplementary Table 3, Fig. 2A).

Differential abundance analysis at the species level revealed 371 differentially abundant taxa (false discovery rate (FDR) < 0.25) between the IgAN patients and healthy controls, with *Absicoccus sp000434355* (coef. = 3.69, FDR = 3.53E-13), *CAG:302 sp001916775* (coef. = 3.41, FDR = 6.08E-04) and *Bacteroides ndongoniae* (coef. = 3.28, FDR = 4.59E-08) showing higher abundance, while *Eubacterium R sp000433975* (coef. = −3.28, FDR = 9.09E-07), and *CAG:462 sp900291465* (coef. = −3.23, FDR = 4.90E-09), *Olsenella E sp900540955* (coef. = −2.98, FDR = 1.75E-03) and *Butyricicoccus A sp002395695* (coef. = −2.52, FDR = 5.00E-08) had a lower abundance in IgAN patients (Fig. 2B and C; Supplementary Table 4).

The functional consequences derived from gene mapping were supported by comparative MetaCyc metabolic pathway profiling between the gut metagenomic sample pools of control subjects and IgAN patients, revealing 34 significantly differentially abundant gut microbiome functional pathways. 4-Hydroxyphenylacetate degradation was the most enriched pathway in healthy controls compared with that in IgAN patients (coef. = −3.97, FDR = 1.00E-01), followed by the superpathway of lipopolysaccharide biosynthesis (coef. = −3.53, FDR = 2.24E-01) and sulfoquinovose degradation I pathway (coef. = −3.51, FDR = 1.43E-01). However, multiple nucleotide- and nucleoside-biosynthesis-related pathways (including adenosine, guanosine, inosine, and pyrimidine biosynthesis) were more prominent in IgAN patients, as was glycolysis from glucose-6-phosphate (Fig. 2D, Supplementary Table 5).

Factors affecting the variation in the abundance of gut microbiome functional pathways were serum levels of Gd-IgA1 (R² = 0.06; p-value = 1.60E-03), eGFR (R² = 0.04; p-value = 1.53E-02), and BMI (R² = 0.04; p-value = 2.36E-02) (Supplementary Table 6). According to MaAsLin2 analysis, Gd-IgA1 levels were significantly associated with 58 metabolic pathways, with 37 showing positive associations (e.g., myristic acid biosynthesis in mitochondria pathway: coef. = 1.64; FDR = 5.84E-02) and 20 showing negative associations (e.g., dTDP-N-acetylviosamine biosynthesis: coef. = −1.06; FDR = 1.18E-01) (Supplementary Table 7).

### Comparative analysis of the gut microbiome composition between progressors and non-progressors

**Fig. 3:**
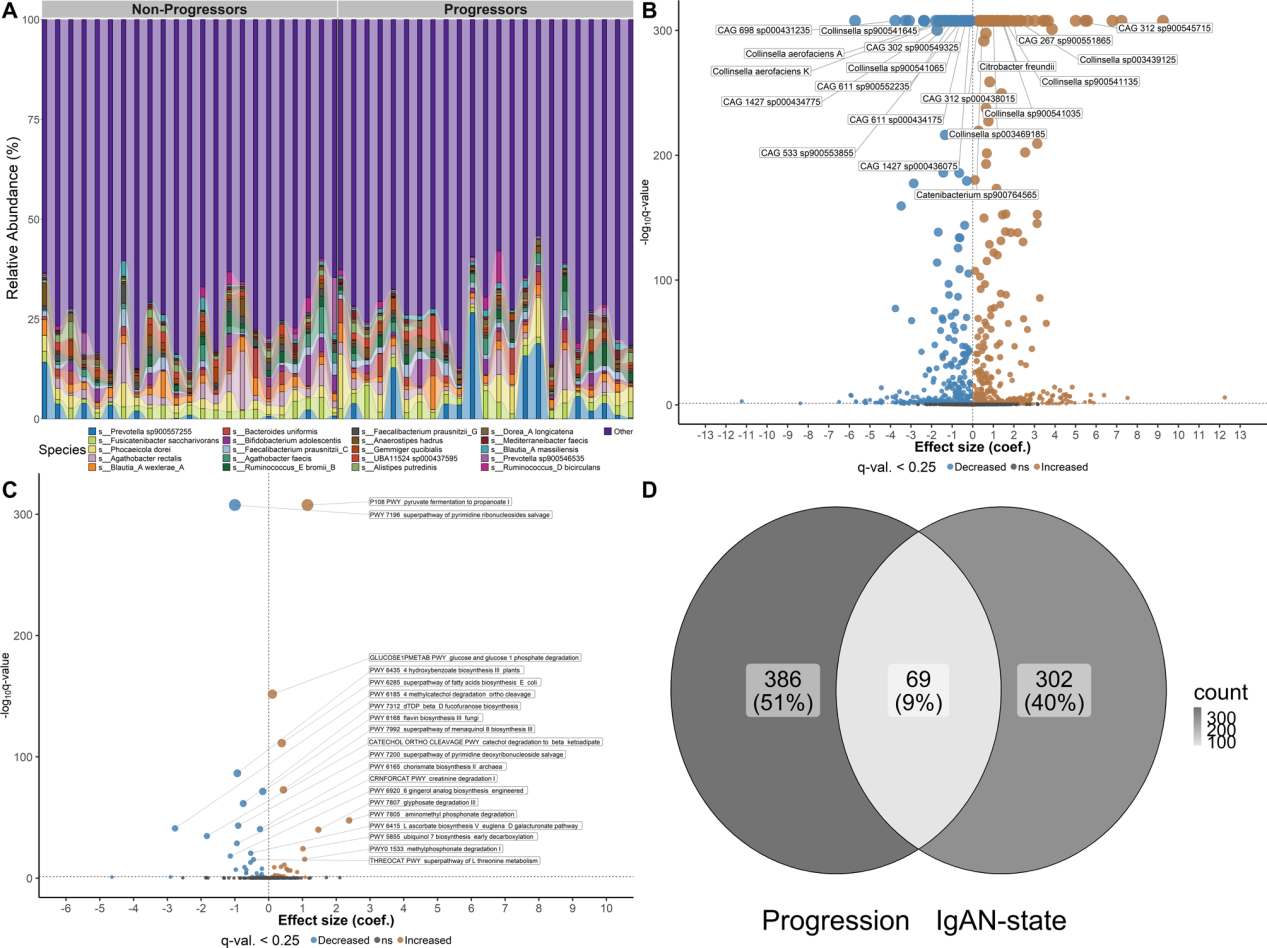
Differences in microbiome composition between IgAN progressors and nonprogressors. **A** Taxonomy bar plot depicting the 20 most representative species for each individual, stratified by study group. **B** Volcano plot representing the relationship between the effect size (Maaslin2 coefficient) and FDR between IgAN progressors and nonprogressors. Labeled values represent the top 20 species based on FDR. A positive coefficient (brown) represents taxa with increased abundance in the progressor group, and a negative coefficient (blue) represents taxa with increased abundance in the nonprogressor group. Model adjusted for BMI. **C** Volcano plot of MetaCyc metabolic differential pathways. The effect size is expressed as a coefficient with positive values for pathways enriched in IgAN progressors and negative values for nonprogressor-related pathways. Labels show top 20 enriched pathways based on FDR. Model adjusted for Gd-IgA1 and BMI. **D** Venn diagram depicting the overlapping species identified from the differential abundance analyses of the case–control and progressor–nonprogressor comparisons. **C** and **D** FDR values of 0 were replaced with the smallest non-zero normalized floating-point number in the R statistical programming language (2.225074e-308), to allow for logarithmic transformation and visualization. Covariates included in the models were selected based on association with microbiome variation as determined by PERMANOVA analysis.

Next, we aimed to assess the potential differences in the gut microbiome composition by focusing solely on the patient group. We retrospectively stratified the patients into two groups based on their disease progression status: 23 were progressors with an eGFR decrease of more than 5 ml/min per year, and 23 were nonprogressors (Supplementary Table 8). The mean follow-up time was 24 months (ranging from 2 to 43 months), with a mean eGFR reduction of 5.8 ml/min/year (ranging from a decline of 30 ml/min/year to an improvement in kidney function by 9 ml/min/year). There was no statistically significant difference in the alpha diversity metrics between the two groups (Supplementary Table 9, Supplementary Fig. 3). Nevertheless, distinct beta diversity clustering was observed between the two groups according to the PERMANOVA test (R^2^ = 0.03; *p* = 0.04), with notably greater dispersion in progressors (Supplementary Table 10, Supplementary Fig. 4). While *Prevotella*, *Faecalibacterium*, and *Blautia* were the most prevalent genera in both groups, the order of abundance differed between them. In progressors, *Prevotella* was the most abundant genus (9.48%), followed by *Blautia* (6.50%) and *Faecalibacterium* (6.12%), whereas *Faecalibacterium* (7.33%) was the most common genus in nonprogressors. At the species level, *Prevotella sp*0*0900557255* was the most abundant in progressors, with *Phocaeicola dorei* and *Fusicatenibacter saccharivorans* also prevalent. Among the nonprogressors, *Agathobacter rectalis* was the most abundant species (2.67%), followed by *Fusicatenibacter saccharivorans* (2.18%) and *Phocaeicola dorei* (2.02%) (Fig. 3A, Supplementary Table 11). The differential abundance analysis at the species level comparing the gut microbiome compositions of progressors and nonprogressors revealed statistically significant differences in 455 taxa. Notably, *Staphylococcus hominis* (coef. = −8.39, FDR = 5.31E-02) and *UBA7173 sp900548705* (coef. = −6.50, FDR = 4.18E-03) were significantly more abundant in nonprogressors, whereas *Dialister hominis* (coef. = 6.38, FDR = 1.85E-08) was significantly more abundant in progressors (Fig. 3B, Supplementary Table 12). Among the 455 progression-related taxa, 69 showed differential abundance also in the comparison of controls versus IgAN patients (e.g., *Akkermansia sp004167605*,* Dialister hominis*) (Fig. 3D).

Differential pathway analysis between nonprogressors and progressors revealed 21 metabolic pathways with the N-acetyl D glucosamine biosynthesis II pathway (coef. = −4.64, FDR = 1.27E-01), showing the strongest enrichment in nonprogressors. Progressors exhibited more active isopropanol biosynthesis (coef. = 1.08, FDR = 1.67E-1), followed by biotin biosynthesis II (coef. = 0.52, FDR = 2.28E-1) and phospholipid biosynthesis (Fig. 3C, Supplementary Table 13). Consistent with the case-control comparison, Gd-IgA1 accounted for a larger proportion of the observed variations in metabolic pathways (PERMANOVA R² = 0.06, *p* = 0.018) specifically among IgAN patients (Supplementary Table 14). Furthermore, in IgAN patients only, serum Gd-IgA1 levels were significantly associated with the abundance of 88 metabolic pathways, including 57 positively associated pathways—such as phospholipid remodeling in yeast (coef. = 1.97; FDR = 1.17E-01)—and 31 negatively associated pathways, such as the superpathway of UDP-N-acetylglucosamine-derived O-antigen building blocks biosynthesis (coef. = −0.69; FDR = 4.33E-29) or creatinine degradation I (coef. = −0.67; FDR = 3.03E-54) (Supplementary Table 15).

## Discussion

Our findings align with previous gut microbiome studies in the Latvian population, which have identified *Prevotella* as the most common genus, showing a similar microbial community composition between the IgAN and HC groups. The high consumption of a plant-based, high-fiber diet is plausibly the primary reason for the high abundance of *Prevotella*. Interestingly, the data from our cohort in Latvia are like those of non-western populations (India, Peru, Tanzania, and Madagascar)^25^. In contrast, the gut microbiome of Western populations, such as those in the United States and Spain, which consume a typical Westernized diet (rich in protein and fat and low in plant-based fibres), is enriched mainly in *Bacteroides* and *Ruminococcus*, with a very low abundance of *Prevotella*^26^. According to a recent meta-analysis of gut microbiome characteristics in IgAN patients, which involved 11 cross-sectional studies and included 652 individuals, no consistent significant variations in the abundance of specific bacteria were noted across the different studies. For example, the *Bacteroides* genus had a greater relative abundance in patients with IgAN than in healthy individuals in three studies, a lower abundance in three studies, and no significant differences in four studies. Similarly, ambiguous data were found for *Streptococcus*, with higher abundances in patients with IgAN in two studies, lower abundances in one study, and no significant changes in eight studies^8^.

The differential abundance analysis in our study revealed that *Butyrococcus* and *Eubacterium rectale* (*Agathobacter rectalis*) were less abundant in IgAN patients than in healthy controls. Both bacteria produce butyrate from carbohydrates^27^. *E.rectale* preferentially colonizes the mucus layer, thereby enhancing the bioavailability of butyrate for colon epithelial cells^28^. Butyrate promotes intestinal barrier function by strengthening tight junctions and producing mucin and antimicrobial peptides and is essential for the differentiation of CD4-positive regulatory T cells (Tregs), which suppress immune responses^29,30^. According to Yang et al., the number of Tregs is decreased in IgAN patients^31^. Moreover, *Agathobacter rectalis* was the most prevalent bacteria in nonprogressors, patients with a favourable prognosis. These results highlight the potential importance of butyrate substitutions or dietary interventions. There are no other studies showing a greater abundance of *Bacteroides ndongoniae* in kidney disease; nevertheless, the *Bacteroides* genus could be present in greater quantities in patients with end-stage kidney disease^32^.

We found no significant difference in gut microbial diversity between IgAN patients and healthy individuals. These findings suggest that not the gut microbiome composition or microbial diversity plays a crucial role in IgAN pathogenesis but, hypothetically, changes in microbial community function.

Increased nucleoside (adenosine, guanosine, and inosine) biosynthesis pathways were found in IgAN patients. Extracellular adenosine is increased during intestinal inflammation. A key contributing factor to the development of intestinal inflammation is intestinal barrier dysfunction, which is associated with an altered microbiome composition, bacterial infiltration, impaired cell junctions, loss of the mucus layer and acidification. This contributes to the release of ATP from injured cells, which is converted to the alarm molecule adenosine^33^. Adenosine is a potent endogenous anti-inflammatory agent that regulates the function of inflammatory cells via interaction with specific receptors expressed on lymphocytes, inhibiting Th17 differentiation and stimulating Treg differentiation^34^. Inosine is formed from the breakdown of adenosine and has been shown to exert powerful anti-inflammatory effects related to the activation of adenosine receptors^35^.

Guanosine is a fundamental component of nucleotides and is essential for DNA and RNA synthesis^36^. These processes are vital in rapidly dividing cells, such as immune cells (e.g., lymphocytes and plasma cells), which require substantial amounts of nucleotides to support their proliferation during immune response. Lymphocytes and plasma cells are highly metabolically active during activation and expansion, as they produce antibodies and mediate immune responses. The increased demand for guanosine and other purine nucleotides in these cells aligns with their role in immune activation and inflammation, particularly in conditions such as IgAN, where aberrant immune responses are implicated. Another pathway that is involved in the proliferative and inflammatory process, glycolysis from glucose 6-phosphate, was also increased in IgAN patients compared with healthy controls. Glycolysis is typically used as an energy source and is known to be upregulated in dividing cells^37^.

The 4-hydroxyphenylacetate degradation pathway was more prominent in healthy controls than in IgAN patients. 4-Hydroxyphenylacetate is a common product during the fermentation of aromatic amino acids, particularly in the L-tyrosine degradation pathway, which is carried out by several *Escherichia coli* strains and generates pyruvate and succinate as end products^38^. Higher succinate production by the gut microbiota is promoted by a high-protein diet^39^. Usually, chronic kidney disease patients are recommended to decrease protein intake in their diet, resulting in potentially increased protein consumption in healthy individuals.

Another pathway that was decreased in IgAN patients—was the sulfoquinovose degradation I pathway. Sulfoquinovose is a plant-derived sulfonated monosaccharide that serves as the polar headgroup of the sulfolipids in photosynthetic membranes. The final product of this pathway is glycerone phosphate, which powers the cell via glycolysis from glucose 6-phosphate, and 2,3-dihydroxypropane-1-sulfonate in most individuals fermented mainly by *E. rectale*^40^. Afterwards, it is excreted and can be further degraded by other bacteria and serves as a source of sulfite^41^. As mentioned previously, *E.rectale* in our study was less abundant in IgAN patients, which confirms the possibility of delayed sulfoquinovose degradation. To date, there are no other studies in IgAN patients showing this pathway alteration.

Previous studies have analysed the bacterial composition in progressors and nonprogressors. Nonprogressor patients were defined as having a 50% decrease in daily proteinuria and stable kidney function (eGFR) after six months compared with baseline (De Angelis); however, metabolic pathways were not studied between the two groups. In our study, the progression rate was evaluated according to a significant GFR slope of 5 ml/min/1.73 m^2^/year at a mean follow-up time of 24 months, which is usually irreversible. Pathways enriched in progressors were connected to phospholipid synthesis in bacteria, which are essential components of bacterial cell membranes. Gram-negative bacteria have a cell envelope composed of three distinct layers: the inner membrane, a periplasmic space containing the peptidoglycan cell wall, and the outer membrane, with its inner leaflet containing phospholipids and its outer leaflet predominantly composed of lipopolysaccharides^42^. Phospholipid synthesis may be adapted to increase bacterial survival, colonization, or competition.

The main limitation of this study is its small sample size, which restricts confounder control and the detection of less prevalent microbial taxa. Additionally, while we employed a data-driven approach—using PERMANOVA—to select covariates for differential abundance and pathway analyses, we acknowledge that this method does not account for potential unmeasured confounders. Factors not captured in our dataset, such as dietary patterns, medication use, or environmental exposures, could influence microbiome composition and function and thus may impact our findings. Nevertheless, the study benefits from a well-characterized patient cohort. As a noninterventional study, it does not establish causal relationships. We fully acknowledge that metabolic pathways inferred from bacterial DNA through metagenomic analysis represent only the putative functional potential of the microbiome, rather than direct evidence of metabolite activity. In our study, we employed established bioinformatics tools such as HUMAnN3, which leverage curated databases to predict pathway abundances from metagenomic content. While this approach is widely accepted in microbiome research and offers valuable insights into potential functional alterations, it does not provide confirmation of actual metabolite production or activity. Given the strong evidence of IgAN-related shifts in gut microbiome functions, future studies may benefit from integrating metabolomics or metatranscriptomics data to provide deeper insights into the functional consequences of these microbial alterations.

The renoprotective effects of butyrate have been observed in various types of kidney diseases: in cisplatin-induced kidney injury^43^ diabetic kidney disease^44^ obesity-related glomerulopathy^45^ septic kidney injury modeled by lipopolysaccharide administration. In the latter model, sodium butyrate exerted nephroprotective effects by modulating Toll-like receptor (TLR) 2/4 signaling to regulate β-defensin 2 expression, thereby reducing inflammation^46^. Another proposed mechanism involves the inhibition of both pyroptosis and apoptosis^47^. As lipopolysaccharides derived from gut bacteria contribute to IgAN pathogenesis by promoting IgA1 overproduction and hypogalactosylation through TLR4 activation^48^ and given the decreased abundance of butyrate-producing bacteria observed in IgAN patients, future studies should investigate the potential therapeutic benefits of butyrate supplementation in IgAN management.

We have reported associations between specific microbial taxa in IgAN patients compared to healthy individuals, as well as in relation to kidney function decline. However, such associations do not imply causation. It remains essential to determine whether these microbiome alterations are a causal factor in IgAN development or a consequence of the disease process. A combination of advanced computational approaches, animal models, and clinical trials is essential to clarify the role of gut microbiome alterations in disease pathogenesis. These efforts should also account for the critical influence of diet on the gut–kidney axis.

## Conclusions

Our study suggests that the metabolic pathways and functional activity of the gut microbiome, rather than its composition alone, play a significant role in IgAN. Notably, no significant differences in microbial community diversity were found between IgAN patients and healthy controls. While IgAN patients exhibit a reduction in butyrate-producing bacteria, which are essential for maintaining intestinal barrier function, functional profiling points to immune activation and inflammation. Additionally, there is evidence of potential compensatory mechanisms, such as the stabilization of bacterial cellular membranes in the IgAN progressor group.

## Supplementary Information

Below is the link to the electronic supplementary material.

Supplementary Material 1.

## Data Availability

Clinical and metagenomic data have been submitted to the European Nucleotide Archive repository with the primary accession code PRJEB85648.
