# Supplementary material for: Reduced butyrate-producing bacteria and altered metabolic pathways in the gut microbiome of immunoglobulin A nephropathy patients
Source: Sci Rep. 2025 Jul 31;15:28011. doi: 10.1038/s41598-025-13629-5 (PMC12313972; doi:10.1038/s41598-025-13629-5)
Supplement: Supplementary file 1 — Supplementary Material 1. [file 41598_2025_13629_MOESM1_ESM.docx]

**Reduced butyrate-producing bacteria and altered metabolic pathways in the gut microbiome of immunoglobulin A nephropathy patients**

**Additional supplementary files**

**Supplementary Table 1.** Comparison of various alpha diversity measures between IgAN patients and healthy controls (Wilcoxon test, Benjamini-Hochberg procedure).

| **Method** | **IgAN cases (median, IQR)** | **Controls (median, IQR)** | **p** | **p.adj** |
| --- | --- | --- | --- | --- |
| Observed taxa | 2042.50 (1900.75 - 2120.25) | 2044.00 (1919.00 - 2181.00) | 0.7 | 7.00E-01 |
| Pielou's evenness | 0.65 (0.62 - 0.68) | 0.65 (0.64 - 0.69) | 0.3 | 3.10E-01 |
| Shannon index | 4.92 (4.72 - 5.19) | 5.01 (5.25 - 4.79) | 0.4 | 3.60E-01 |
| Inverse Simpson index | 54.88 (78.21 - 39.50) | 61.30 (82.88 - 50.55) | 0.5 | 4.50E-01 |
| Chao1 | 2042.50 (1900.75 - 2120.25) | 2044.00 (1919.00 - 2181.00) | 0.7 | 7.00E-01 |


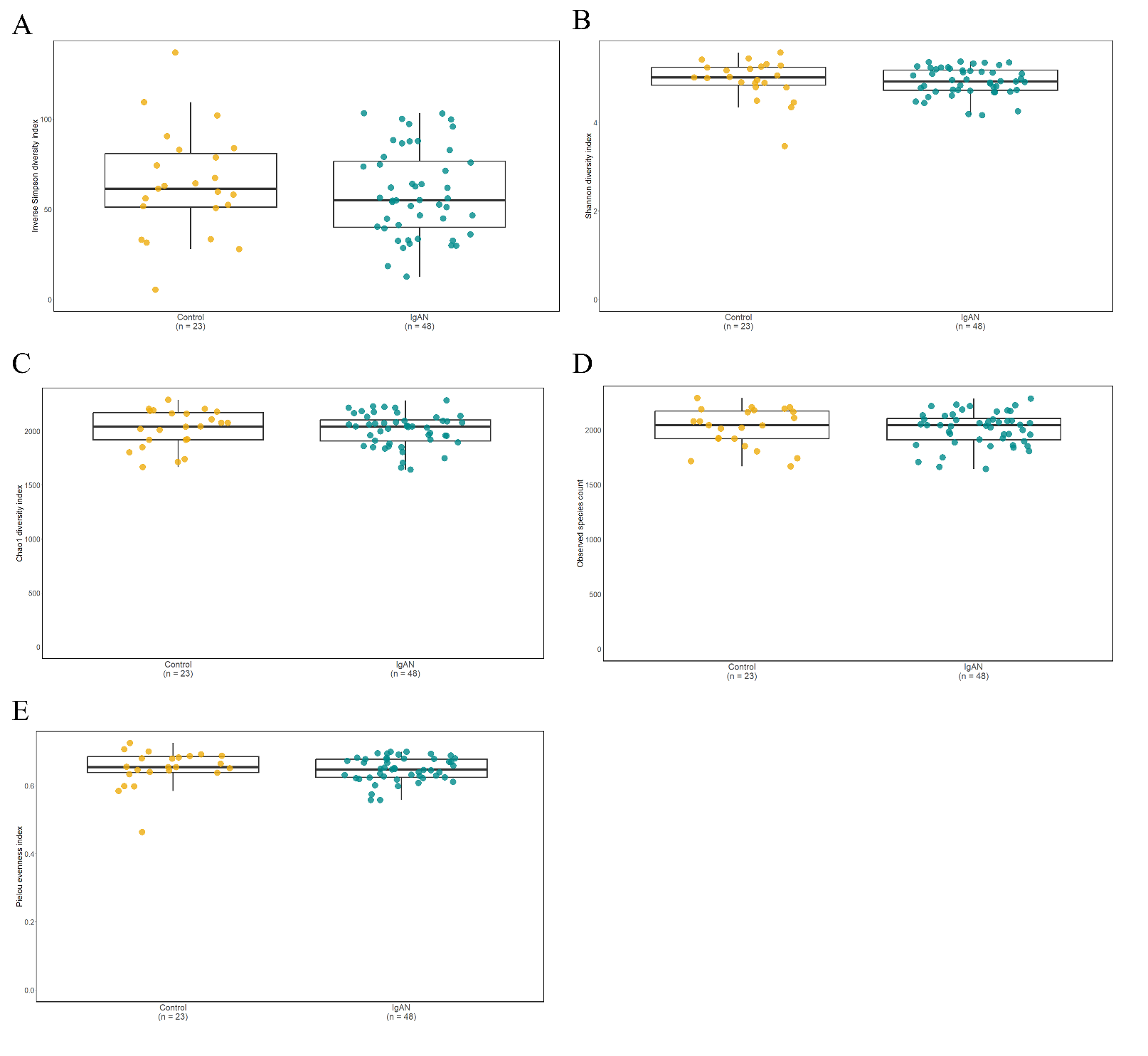


**Supplementary Figure 1. Alpha diversity measures of IgA nephropathy patients and healthy controls**. A: Inverse Simpson diversity index; B: Shannon diversity index; C: Chao1 diversity index; D: Observed taxa; E: Pielou's evenness index. Boxplots present the median, 25th, and 75th percentiles.

**Supplementary Table 2.** Contributing factors determining the inter-sample variation of gut microbiome profiles in IgA nephropathy patients and healthy controls, as identified by PERMANOVA.

|  | **Df** | **Sum Of Sqs** | **R2** | **F** | **Pr(>F)** |
| --- | --- | --- | --- | --- | --- |
| Indoxylsulphate, ng/ml | 1 | 0.20 | 0.01 | 0.95 | 5.24E-01 |
| Sex | 1 | 0.21 | 0.02 | 1.02 | 3.97E-01 |
| BMI, kg/m^2^ | 1 | 0.28 | 0.02 | 1.33 | 8.20E-02 |
| eGFR, ml/min/1,73m^2^ | 1 | 0.27 | 0.02 | 1.28 | 1.03E-01 |
| Lipopolysaccharides, pg/ml | 1 | 0.22 | 0.02 | 1.08 | 3.08E-01 |
| Gd-IgA1, ng/ml | 1 | 0.16 | 0.01 | 0.77 | 8.78E-01 |
| Type | 1 | 0.22 | 0.02 | 1.04 | 3.55E-01 |
| Residual | 60 | 12.45 | 0.89 | NA | NA |
| Total | 67 | 13.96 | 1.00 | NA | NA |
|  |  |  |  |  |  |
|  |  |  |  |  |  |


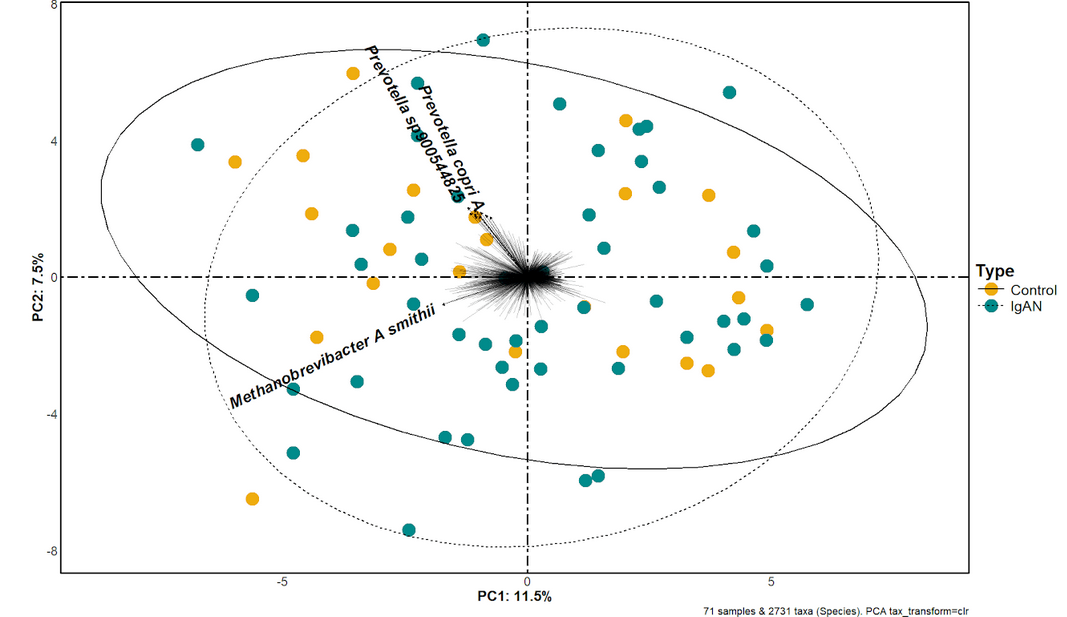
**Supplementary Figure 2.** Principal component analysis plot showing inter-sample variability in microbial community composition among IgA nephropathy patients and healthy controls.

**Supplementary Table 3.** The absolute and relative abundances of gut microbiome taxa at the species level in IgA nephropathy patients and healthy controls.

| **Species** | **Class** | **Type** | **Abundance by type** | **Relative abundance by type** |
| --- | --- | --- | --- | --- |
| s__Prevotella sp900557255 | Species | IgAN | 28187777 | 2.95 |
| s__Prevotella sp900557255 | Species | Control | 20211633 | 4.34 |
| s__Fusicatenibacter saccharivorans | Species | IgAN | 24163363 | 2.53 |
| s__Fusicatenibacter saccharivorans | Species | Control | 12002234 | 2.58 |
| s__Phocaeicola dorei | Species | IgAN | 23929204 | 2.51 |
| s__Phocaeicola dorei | Species | Control | 10609345 | 2.28 |
| s__Agathobacter rectalis | Species | IgAN | 22036751 | 2.31 |
| s__Agathobacter rectalis | Species | Control | 11600949 | 2.49 |
| s__Blautia_A wexlerae_A | Species | IgAN | 18832342 | 1.97 |
| s__Blautia_A wexlerae_A | Species | Control | 8734886 | 1.87 |
| s__Bacteroides uniformis | Species | IgAN | 16211483 | 1.7 |
| s__Bacteroides uniformis | Species | Control | 7703844 | 1.65 |
| s__Bifidobacterium adolescentis | Species | IgAN | 12496400 | 1.31 |
| s__Bifidobacterium adolescentis | Species | Control | 5350744 | 1.15 |
| s__Faecalibacterium prausnitzii_C | Species | IgAN | 11762986 | 1.23 |
| s__Faecalibacterium prausnitzii_C | Species | Control | 5880692 | 1.26 |
| s__Agathobacter faecis | Species | IgAN | 11201421 | 1.17 |
| s__Agathobacter faecis | Species | Control | 5925322 | 1.27 |
| s__Faecalibacterium prausnitzii_G | Species | IgAN | 9860898 | 1.03 |
| s__Faecalibacterium prausnitzii_G | Species | Control | 6910298 | 1.48 |
| s__Ruminococcus_E bromii_B | Species | IgAN | 10213089 | 1.07 |
| s__Ruminococcus_E bromii_B | Species | Control | 4802896 | 1.03 |
| s__Gemmiger qucibialis | Species | IgAN | 9021516 | 0.94 |
| s__Gemmiger qucibialis | Species | Control | 5243614 | 1.13 |
| s__Anaerostipes hadrus | Species | IgAN | 9487240 | 0.99 |
| s__Anaerostipes hadrus | Species | Control | 3206939 | 0.69 |
| s__Blautia_A massiliensis | Species | IgAN | 8006770 | 0.84 |
| s__Blautia_A massiliensis | Species | Control | 3874215 | 0.83 |
| s__Dorea_A longicatena | Species | IgAN | 7816084 | 0.82 |
| s__Dorea_A longicatena | Species | Control | 4026839 | 0.86 |
| s__Alistipes putredinis | Species | IgAN | 7829006 | 0.82 |
| s__Alistipes putredinis | Species | Control | 3831679 | 0.82 |
| s__KLE1615 sp900066985 | Species | IgAN | 6819088 | 0.71 |
| s__KLE1615 sp900066985 | Species | Control | 3959725 | 0.85 |
| s__Faecalibacterium prausnitzii_D | Species | IgAN | 5570673 | 0.58 |
| s__Faecalibacterium prausnitzii_D | Species | Control | 5201596 | 1.12 |
| s__Blautia_A faecis | Species | IgAN | 7184637 | 0.75 |
| s__Blautia_A faecis | Species | Control | 3507104 | 0.75 |
| s__Faecalibacterium prausnitzii | Species | IgAN | 6506901 | 0.68 |
| s__Faecalibacterium prausnitzii | Species | Control | 4170570 | 0.89 |
| Others | Species | IgAN | 697963837 | 73.08 |
| Others | Species | Control | 329314957 | 70.66 |

**Supplementary Table 4.** Differentially abundant species comparing IgA nephropathy patients and healthy controls.

| **Feature** | **coef** | **stderr** | **pval** | **qval** |
| --- | --- | --- | --- | --- |
| Absicoccus sp000434355 | 3.69 | 0.48 | 1.10E-14 | 3.53E-13 |
| CAG:302 sp001916775 | 3.41 | 0.83 | 3.89E-05 | 6.08E-04 |
| Bacteroides ndongoniae | 3.28 | 0.55 | 1.80E-09 | 4.59E-08 |
| Eubacterium R sp000433975 | -3.28 | 0.60 | 3.73E-08 | 9.09E-07 |
| CAG:462 sp900291465 | -3.23 | 0.51 | 1.82E-10 | 4.90E-09 |
| Olsenella E sp900540955 | -2.98 | 0.78 | 1.26E-04 | 1.75E-03 |
| CAG:433 sp900546245 | 2.96 | 0.82 | 3.15E-04 | 3.89E-03 |
| CAG:475 sp900547975 | 2.88 | 0.73 | 7.63E-05 | 1.12E-03 |
| Akkermansia muciniphila C | 2.76 | 0.72 | 1.18E-04 | 1.66E-03 |
| CAG:460 sp000437355 | 2.75 | 1.03 | 7.58E-03 | 5.35E-02 |
| Massilistercora timonensis | 2.73 | 0.48 | 1.11E-08 | 2.80E-07 |
| CAG:115 sp000432175 | -2.68 | 0.55 | 8.48E-07 | 1.79E-05 |
| CAG:245 sp900552135 | 2.61 | 0.46 | 1.56E-08 | 3.87E-07 |
| Butyricicoccus A sp002395695 | -2.52 | 0.42 | 1.97E-09 | 5.00E-08 |
| UBA7173 sp900546835 | -2.46 | 0.55 | 6.37E-06 | 1.17E-04 |
| CAG:196 sp002102975 | 2.46 | 0.76 | 1.22E-03 | 1.24E-02 |
| Bacteroides togonis | 2.46 | 0.50 | 1.10E-06 | 2.27E-05 |
| UMGS1901 sp900553755 | 2.45 | 0.49 | 6.53E-07 | 1.40E-05 |
| Akkermansia sp004167605 | 2.45 | 0.75 | 1.16E-03 | 1.19E-02 |
| UMGS403 sp900541565 | -2.40 | 0.33 | 2.83E-13 | 8.46E-12 |
| Lacticaseibacillus rhamnosus | -2.32 | 0.69 | 7.57E-04 | 8.30E-03 |
| UBA1777 sp900546515 | -2.30 | 0.30 | 1.39E-14 | 4.40E-13 |
| UMGS363 sp900543105 | -2.19 | 0.42 | 2.05E-07 | 4.61E-06 |
| UBA5394 sp003150565 | 2.15 | 0.81 | 8.12E-03 | 5.63E-02 |
| Enterocloster sp001517625 | -2.13 | 0.32 | 4.56E-11 | 1.26E-09 |
| CAG:510 sp000434615 | -2.10 | 0.50 | 2.86E-05 | 4.60E-04 |
| CAG:115 sp003507295 | 2.10 | 0.48 | 1.37E-05 | 2.36E-04 |
| Parabacteroides sp000436495 | -2.07 | 0.63 | 1.08E-03 | 1.12E-02 |
| Prevotella sp900543975 | 2.06 | 0.54 | 1.24E-04 | 1.73E-03 |
| Akkermansia muciniphila B | 2.05 | 0.68 | 2.70E-03 | 2.38E-02 |
| UBA1777 sp900754515 | 2.05 | 0.39 | 1.62E-07 | 3.69E-06 |
| Streptococcus anginosus C | 1.98 | 0.53 | 2.11E-04 | 2.70E-03 |
| Eubacterium R sp003526845 | 1.97 | 0.50 | 8.00E-05 | 1.17E-03 |
| CAG:495 sp001917125 | -1.94 | 0.78 | 1.30E-02 | 8.15E-02 |
| Ruminococcus F champanellensis | -1.93 | 0.29 | 1.62E-11 | 4.58E-10 |
| RC9 sp000434935 | -1.86 | 0.73 | 1.04E-02 | 6.89E-02 |
| Longicatena caecimuris | 1.85 | 0.42 | 1.06E-05 | 1.87E-04 |
| UBA1394 sp900554975 | 1.84 | 0.45 | 3.67E-05 | 5.78E-04 |
| Phocaeicola barnesiae | -1.84 | 0.50 | 2.52E-04 | 3.19E-03 |
| RC9 sp000435075 | 1.76 | 0.65 | 6.54E-03 | 4.79E-02 |
| UMGS1518 sp900552575 | -1.75 | 0.33 | 1.17E-07 | 2.76E-06 |
| Agathobacter sp900543445 | -1.75 | 0.32 | 5.63E-08 | 1.34E-06 |
| CAG:177 sp000431775 | 1.74 | 0.54 | 1.35E-03 | 1.36E-02 |
| Prevotella sp002265625 | 1.71 | 0.53 | 1.18E-03 | 1.21E-02 |
| CAG:303 sp000437755 | 1.69 | 0.42 | 4.78E-05 | 7.32E-04 |
| UBA9506 sp003506415 | 1.68 | 0.77 | 2.79E-02 | 1.39E-01 |
| UBA11471 sp900542765 | 1.68 | 0.54 | 1.87E-03 | 1.79E-02 |
| UBA10677 sp900762945 | -1.67 | 0.75 | 2.67E-02 | 1.34E-01 |
| UBA10677 sp003533505 | -1.67 | 0.72 | 1.98E-02 | 1.07E-01 |
| CAG:110 sp900540635 | -1.65 | 0.47 | 4.87E-04 | 5.76E-03 |
| TF01 11 sp001916135 | -1.64 | 0.36 | 5.30E-06 | 9.89E-05 |
| Faecalibacterium sp900540455 | -1.64 | 0.26 | 4.18E-10 | 1.09E-08 |
| Prevotella rara | -1.64 | 0.52 | 1.72E-03 | 1.69E-02 |
| CAG:345 sp000433315 | -1.63 | 0.79 | 3.98E-02 | 1.79E-01 |
| Prevotella sp900552675 | 1.62 | 0.59 | 5.75E-03 | 4.33E-02 |
| UMGS172 sp900539855 | 1.60 | 0.47 | 6.50E-04 | 7.34E-03 |

**Supplementary Table 5.** Bacterial metabolic pathways showing different representations between IgA nephropathy patients and healthy controls.

| **Feature** | **coef** | **stderr** | **pval** | **qval** |
| --- | --- | --- | --- | --- |
| PWY.7269..mitochondrial.NADPH.production..yeast. | -4.24 | 1.22 | 4.98E-04 | 5.92E-03 |
| X3.HYDROXYPHENYLACETATE.DEGRADATION.PWY..4.hydroxyphenylacetate.degradation | -3.97 | 1.61 | 1.35E-02 | 1.00E-01 |
| GLYCOLYSIS.TCA.GLYOX.BYPASS..superpathway.of.glycolysis..pyruvate.dehydrogenase..TCA..and.glyoxylate.bypass | -3.92 | 1.23 | 1.40E-03 | 1.59E-02 |
| PWY.7409..phospholipid.remodeling..phosphatidylethanolamine..yeast. | -3.6 | 1.64 | 2.84E-02 | 1.62E-01 |
| LPSSYN.PWY..superpathway.of.lipopolysaccharide.biosynthesis | -3.53 | 1.77 | 4.60E-02 | 2.24E-01 |
| PWY.7446..sulfoquinovose.degradation.I | -3.51 | 1.55 | 2.37E-02 | 1.43E-01 |
| PWY.6328..L.lysine.degradation.X | -3.43 | 1.31 | 8.76E-03 | 7.56E-02 |
| ARGDEG.PWY..superpathway.of.L.arginine..putrescine..and.4.aminobutanoate.degradation | -3.28 | 1.69 | 5.21E-02 | 2.44E-01 |
| ORNARGDEG.PWY..superpathway.of.L.arginine.and.L.ornithine.degradation | -3.28 | 1.69 | 5.21E-02 | 2.44E-01 |
| THREOCAT.PWY..superpathway.of.L.threonine.metabolism | -3.22 | 1.34 | 1.63E-02 | 1.12E-01 |
| PWY.5656..mannosylglycerate.biosynthesis.I | -3.05 | 1.13 | 6.94E-03 | 6.21E-02 |
| CARNMET.PWY..L.carnitine.degradation.I | -2.83 | 1.37 | 3.84E-02 | 1.95E-01 |
| PWY.5855..ubiquinol.7.biosynthesis..early.decarboxylation. | -2.78 | 1.06 | 8.86E-03 | 7.62E-02 |
| PWY0.1221..putrescine.degradation.II | -2.72 | 1.36 | 4.51E-02 | 2.21E-01 |
| AST.PWY..L.arginine.degradation.II..AST.pathway. | -2.62 | 1.08 | 1.58E-02 | 1.10E-01 |
| PWY0.1338..polymyxin.resistance | -2.6 | 1.13 | 2.17E-02 | 1.33E-01 |
| ECASYN.PWY..enterobacterial.common.antigen.biosynthesis | -2.53 | 1.15 | 2.79E-02 | 1.60E-01 |
| PWY.5747..2.methylcitrate.cycle.II | -2.4 | 1.03 | 1.95E-02 | 1.26E-01 |
| PWY.7616..methanol.oxidation.to.carbon.dioxide | -2.36 | 1.13 | 3.64E-02 | 1.88E-01 |
| TCA.GLYOX.BYPASS..superpathway.of.glyoxylate.bypass.and.TCA | -2.27 | 0.96 | 1.78E-02 | 1.20E-01 |
| KDO.NAGLIPASYN.PWY..superpathway.of..Kdo.2.lipid.A.biosynthesis | -2.03 | 0.99 | 3.93E-02 | 1.98E-01 |
| HEME.BIOSYNTHESIS.II.1..heme.b.biosynthesis.V..aerobic. | -1.53 | 0.61 | 1.22E-02 | 9.41E-02 |
| PWY0.1415..superpathway.of.heme.b.biosynthesis.from.uroporphyrinogen.III | -1.52 | 0.76 | 4.52E-02 | 2.21E-01 |
| TCA..TCA.cycle.I..prokaryotic. | -1.5 | 0.76 | 4.89E-02 | 2.33E-01 |
| GLYOXYLATE.BYPASS..glyoxylate.cycle | -1.37 | 0.59 | 2.08E-02 | 1.31E-01 |
| PWY.7094..fatty.acid.salvage | -0.86 | 0.08 | 3.52E-26 | 8.31E-25 |
| SO4ASSIM.PWY..assimilatory.sulfate.reduction.I | -0.8 | 0.31 | 9.37E-03 | 7.93E-02 |
| PWY0.1337..oleate..beta..oxidation | -0.79 | 0.08 | 8.59E-22 | 1.74E-20 |
| PWY.7340..9.cis..11.trans.octadecadienoyl.CoA.degradation..isomerase.dependent..yeast. | -0.77 | 0.14 | 1.02E-08 | 1.56E-07 |
| PWY.5972..stearate.biosynthesis.I..animals. | -0.73 | 0.14 | 3.17E-07 | 4.63E-06 |
| PWY.5464..superpathway.of.cytosolic.glycolysis..plants...pyruvate.dehydrogenase.and.TCA.cycle | -0.69 | 0.1 | 1.51E-12 | 2.51E-11 |
| AEROBACTINSYN.PWY..aerobactin.biosynthesis | -0.69 | 0.21 | 8.83E-04 | 1.03E-02 |
| PWY.5345..superpathway.of.L.methionine.biosynthesis..by.sulfhydrylation. | -0.66 | 0.28 | 1.90E-02 | 1.24E-01 |
| PWY.5088..L.glutamate.degradation.VIII..to.propanoate. | -0.63 | 0.17 | 2.11E-04 | 2.58E-03 |
| SULFATE.CYS.PWY..superpathway.of.sulfate.assimilation.and.cysteine.biosynthesis | -0.63 | 0.28 | 2.67E-02 | 1.56E-01 |
| PWY490.3..nitrate.reduction.VI..assimilatory. | -0.55 | 0.05 | 5.42E-27 | 1.37E-25 |
| PWY.5415..catechol.degradation.I..meta.cleavage.pathway. | -0.53 | 0.27 | 5.26E-02 | 2.45E-01 |
| PWY.6281..L.selenocysteine.biosynthesis.II..archaea.and.eukaryotes. | -0.41 | 0.15 | 5.13E-03 | 4.93E-02 |
| PWY.6922..L.N.delta..acetylornithine.biosynthesis | -0.38 | 0.05 | 5.31E-17 | 1.00E-15 |
| PROPFERM.PWY..superpathway.of.L.alanine.fermentation..Stickland.reaction. | -0.33 | 0.03 | 2.79E-22 | 5.72E-21 |
| PWY.8188..L.alanine.degradation.VI..reductive.Stickland.reaction. | -0.33 | 0.03 | 2.79E-22 | 5.72E-21 |
| PWY.8189..L.alanine.degradation.V..oxidative.Stickland.reaction. | -0.33 | 0.03 | 2.79E-22 | 5.72E-21 |
| PWY.7391..isoprene.biosynthesis.II..engineered. | -0.32 | 0.13 | 1.42E-02 | 1.03E-01 |
| PWY.5494..pyruvate.fermentation.to.propanoate.II..acrylate.pathway. | -0.31 | 0.05 | 1.39E-10 | 2.21E-09 |
| PWY1ZNC.1..assimilatory.sulfate.reduction.IV | -0.3 | 0.15 | 4.27E-02 | 2.11E-01 |
| PWY.7242..D.fructuronate.degradation | -0.28 | 0.11 | 8.93E-03 | 7.62E-02 |
| PWY.6478..GDP.D.glycero..alpha..D.manno.heptose.biosynthesis | -0.26 | 0.1 | 8.61E-03 | 7.46E-02 |
| GLUCUROCAT.PWY..superpathway.of..beta..D.glucuronosides.degradation | -0.24 | 0.09 | 9.06E-03 | 7.70E-02 |
| PWY66.367..ketogenesis | -0.23 | 0.09 | 1.30E-02 | 9.86E-02 |
| GALACTUROCAT.PWY..D.galacturonate.degradation.I | -0.2 | 0.1 | 3.66E-02 | 1.88E-01 |
| PWY.6470..peptidoglycan.biosynthesis.V...beta..lactam.resistance. | -0.19 | 0.09 | 3.79E-02 | 1.93E-01 |
| PWY.6353..purine.nucleotides.degradation.II..aerobic. | -0.16 | 0.07 | 1.89E-02 | 1.24E-01 |
| PWY.6823..molybdopterin.biosynthesis | -0.14 | 0.05 | 7.40E-03 | 6.54E-02 |
| METH.ACETATE.PWY..methanogenesis.from.acetate | -0.14 | 0.06 | 2.49E-02 | 1.49E-01 |
| GLCMANNANAUT.PWY..superpathway.of.N.acetylglucosamine..N.acetylmannosamine.and.N.acetylneuraminate.degradation | -0.14 | 0.06 | 2.68E-02 | 1.56E-01 |
| PWY.7237..myo...chiro..and.scyllo.inositol.degradation | -0.14 | 0.07 | 4.89E-02 | 2.33E-01 |
| PWY0.1296..purine.ribonucleosides.degradation | -0.11 | 0.04 | 6.91E-03 | 6.21E-02 |
| DTDPRHAMSYN.PWY..dTDP..beta..L.rhamnose.biosynthesis | -0.11 | 0.06 | 4.13E-02 | 2.06E-01 |
| PWY.7357..thiamine.phosphate.formation.from.pyrithiamine.and.oxythiamine..yeast. | -0.08 | 0.04 | 2.76E-02 | 1.59E-01 |
| PWY.6285..superpathway.of.fatty.acids.biosynthesis..E..coli. | -0.06 | 0.02 | 3.64E-03 | 3.71E-02 |
| PWY.1042..glycolysis.IV | -0.06 | 0.03 | 3.53E-02 | 1.83E-01 |
| PWY.724..superpathway.of.L.lysine..L.threonine.and.L.methionine.biosynthesis.II | 0.04 | 0.02 | 3.49E-02 | 1.82E-01 |
| SER.GLYSYN.PWY..superpathway.of.L.serine.and.glycine.biosynthesis.I | 0.09 | 0.04 | 1.33E-02 | 9.97E-02 |
| PWY.6703..preQ0.biosynthesis | 0.11 | 0.06 | 5.40E-02 | 2.48E-01 |
| OANTIGEN.PWY..O.antigen.building.blocks.biosynthesis..E..coli. | 0.12 | 0.05 | 1.21E-02 | 9.40E-02 |
| PWY.841..superpathway.of.purine.nucleotides.de.novo.biosynthesis.I | 0.13 | 0.05 | 2.03E-02 | 1.29E-01 |
| PWY.7229..superpathway.of.adenosine.nucleotides.de.novo.biosynthesis.I | 0.14 | 0.06 | 1.66E-02 | 1.13E-01 |
| PWY.6126..superpathway.of.adenosine.nucleotides.de.novo.biosynthesis.II | 0.16 | 0.07 | 2.13E-02 | 1.32E-01 |
| PWY.7345..superpathway.of.anaerobic.sucrose.degradation | 0.17 | 0.07 | 1.34E-02 | 9.97E-02 |
| PWY.7228..superpathway.of.guanosine.nucleotides.de.novo.biosynthesis.I | 0.17 | 0.07 | 1.57E-02 | 1.10E-01 |
| PWY.5392..reductive.TCA.cycle.II | 0.18 | 0.07 | 1.04E-02 | 8.47E-02 |
| PWY.7208..superpathway.of.pyrimidine.nucleobases.salvage | 0.18 | 0.08 | 1.89E-02 | 1.24E-01 |
| PWY.6125..superpathway.of.guanosine.nucleotides.de.novo.biosynthesis.II | 0.18 | 0.08 | 1.91E-02 | 1.24E-01 |
| GLYCOLYSIS.E.D..superpathway.of.glycolysis.and.the.Entner.Doudoroff.pathway | 0.19 | 0.09 | 3.32E-02 | 1.76E-01 |
| GLUCOSE1PMETAB.PWY..glucose.and.glucose.1.phosphate.degradation | 0.2 | 0 | 0.00E+00 | 0.00E+00 |
| PWY.7220..adenosine.deoxyribonucleotides.de.novo.biosynthesis.II | 0.2 | 0.08 | 2.03E-02 | 1.29E-01 |
| PWY.7222..guanosine.deoxyribonucleotides.de.novo.biosynthesis.II | 0.2 | 0.08 | 2.03E-02 | 1.29E-01 |
| PWY.6628..superpathway.of.L.phenylalanine.biosynthesis | 0.21 | 0.07 | 3.48E-03 | 3.57E-02 |
| UDPNAGSYN.PWY..UDP.N.acetyl.D.glucosamine.biosynthesis.I | 0.21 | 0.07 | 4.56E-03 | 4.45E-02 |
| PWY.7197..pyrimidine.deoxyribonucleotide.phosphorylation | 0.21 | 0.08 | 6.01E-03 | 5.65E-02 |
| PWY.8187..L.arginine.degradation.XIII..reductive.Stickland.reaction. | 0.22 | 0.11 | 5.31E-02 | 2.46E-01 |
| ANAEROFRUCAT.PWY..homolactic.fermentation | 0.23 | 0.08 | 3.08E-03 | 3.19E-02 |
| PWY.5677..succinate.fermentation.to.butanoate | 0.23 | 0.08 | 4.16E-03 | 4.14E-02 |
| GLYCOLYSIS..glycolysis.I..from.glucose.6.phosphate. | 0.24 | 0.08 | 3.98E-03 | 4.03E-02 |
| PWY.6901..superpathway.of.glucose.and.xylose.degradation | 0.24 | 0.09 | 1.32E-02 | 9.93E-02 |
| PWY.8004..Entner.Doudoroff.pathway.I | 0.25 | 0.11 | 2.00E-02 | 1.28E-01 |
| PENTOSE.P.PWY..pentose.phosphate.pathway | 0.25 | 0.11 | 2.69E-02 | 1.56E-01 |
| P23.PWY..reductive.TCA.cycle.I | 0.26 | 0.04 | 1.56E-12 | 2.58E-11 |
| PWY.5484..glycolysis.II..from.fructose.6.phosphate. | 0.26 | 0.09 | 2.65E-03 | 2.84E-02 |
| PWY.7200..superpathway.of.pyrimidine.deoxyribonucleoside.salvage | 0.27 | 0.06 | 6.33E-06 | 8.78E-05 |
| PWY0.162..superpathway.of.pyrimidine.ribonucleotides.de.novo.biosynthesis | 0.29 | 0.07 | 3.79E-05 | 4.88E-04 |
| PWY.5384..sucrose.degradation.IV..sucrose.phosphorylase. | 0.3 | 0.12 | 1.19E-02 | 9.34E-02 |
| PWY0.1479..tRNA.processing | 0.32 | 0.11 | 2.48E-03 | 2.69E-02 |
| PWY.1861..formaldehyde.assimilation.II..assimilatory.RuMP.Cycle. | 0.33 | 0.15 | 2.72E-02 | 1.57E-01 |
| PWY.4041...gamma..glutamyl.cycle | 0.34 | 0.11 | 1.94E-03 | 2.16E-02 |
| PWY.7234..inosine.5..phosphate.biosynthesis.III | 0.34 | 0.12 | 5.12E-03 | 4.93E-02 |
| P621.PWY..nylon.6.oligomer.degradation | 0.35 | 0.05 | 9.60E-12 | 1.56E-10 |
| PWY.6282..palmitoleate.biosynthesis.I..from..5Z..dodec.5.enoate. | 0.37 | 0.19 | 4.97E-02 | 2.34E-01 |
| PWY.7664..oleate.biosynthesis.IV..anaerobic. | 0.38 | 0.2 | 5.45E-02 | 2.49E-01 |
| PWY.7198..pyrimidine.deoxyribonucleotides.de.novo.biosynthesis.IV | 0.39 | 0.15 | 9.54E-03 | 7.96E-02 |
| PWY.6168..flavin.biosynthesis.III..fungi. | 0.43 | 0.01 | 0.00E+00 | 0.00E+00 |
| PWY.5994..palmitate.biosynthesis..type.I.fatty.acid.synthase. | 0.47 | 0.03 | 1.18E-48 | 4.44E-47 |
| PWY.702..L.methionine.biosynthesis.II | 0.52 | 0.11 | 5.81E-06 | 8.16E-05 |
| PWY0.862...5Z..dodecenoate.biosynthesis.I | 0.52 | 0.21 | 1.14E-02 | 9.01E-02 |
| P108.PWY..pyruvate.fermentation.to.propanoate.I | 0.55 | 0.03 | 6.16E-91 | 3.23E-89 |
| PWY.241..C4.photosynthetic.carbon.assimilation.cycle..NADP.ME.type | 0.55 | 0.25 | 2.69E-02 | 1.56E-01 |
| PWY.7117..C4.photosynthetic.carbon.assimilation.cycle..PEPCK.type | 0.58 | 0.24 | 1.80E-02 | 1.21E-01 |
| PWY.7992..superpathway.of.menaquinol.8.biosynthesis.III | 0.6 | 0.03 | 7.64E-84 | 3.82E-82 |
| PWY.6165..chorismate.biosynthesis.II..archaea. | 0.65 | 0.02 | 6.21E-233 | 5.56E-231 |
| DAPLYSINESYN.PWY..L.lysine.biosynthesis.I | 0.66 | 0.17 | 8.49E-05 | 1.05E-03 |
| PWY.7209..superpathway.of.pyrimidine.ribonucleosides.degradation | 0.67 | 0.01 | 0.00E+00 | 0.00E+00 |
| PWY.7688..dTDP..alpha..D.ravidosamine.and.dTDP.4.acetyl..alpha..D.ravidosamine.biosynthesis | 0.69 | 0.05 | 9.01E-37 | 2.85E-35 |
| P124.PWY..Bifidobacterium.shunt | 0.69 | 0.28 | 1.20E-02 | 9.40E-02 |
| PWY.I9..L.cysteine.biosynthesis.VI..from.L.methionine. | 0.7 | 0.15 | 3.70E-06 | 5.24E-05 |
| PYRIDNUCSAL.PWY..NAD.salvage.pathway.I..PNC.VI.cycle. | 0.72 | 0.3 | 1.47E-02 | 1.05E-01 |
| PWY3O.4107..NAD.salvage.pathway.V..PNC.V.cycle. | 0.8 | 0.31 | 1.02E-02 | 8.33E-02 |
| PWY.622..starch.biosynthesis | 0.88 | 0.01 | 0.00E+00 | 0.00E+00 |
| PWY.6167..flavin.biosynthesis.II..archaea. | 0.97 | 0.03 | 2.40E-245 | 2.34E-243 |
| PWY.6471..peptidoglycan.biosynthesis.IV..Enterococcus.faecium. | 0.97 | 0.06 | 5.42E-51 | 2.20E-49 |
| PWY.6920..6.gingerol.analog.biosynthesis..engineered. | 1.09 | 0.13 | 3.20E-18 | 6.15E-17 |
| FASYN.INITIAL.PWY..superpathway.of.fatty.acid.biosynthesis.initiation..E..coli. | 1.26 | 0.58 | 3.04E-02 | 1.68E-01 |
| PWY.7196..superpathway.of.pyrimidine.ribonucleosides.salvage | 1.32 | 0.03 | 0.00E+00 | 0.00E+00 |
| PWY.6435..4.hydroxybenzoate.biosynthesis.III..plants. | 1.32 | 0.06 | 5.90E-98 | 3.17E-96 |
| PWY66.430..myristate.biosynthesis..mitochondria. | 2.84 | 1.39 | 4.04E-02 | 2.03E-01 |
| PWY.7388..octanoyl..acyl.carrier.protein..biosynthesis..mitochondria..yeast. | 2.87 | 1.38 | 3.80E-02 | 1.93E-01 |
| PWY.6953..dTDP.3.acetamido..alpha..D.fucose.biosynthesis | 3.55 | 0.29 | 6.05E-34 | 1.86E-32 |

**Supplementary Table 6.** Contributing factors determining the observed variation in the gut microbiome-derived metabolic pathways among IgA nephropathy patients and healthy controls.

| **Variable** | **Df** | **Sum Of Sqs** | **R2** | **F** | **Pr (>F)** |
| --- | --- | --- | --- | --- | --- |
| Gd-IgA1 | 1 | 0.04 | 0.06 | 4.52 | 1.60E-03 |
| eGFR | 1 | 0.03 | 0.04 | 2.93 | 1.53E-02 |
| BMI | 1 | 0.02 | 0.04 | 2.68 | 2.36E-02 |
| Indoxylsulfate | 1 | 0.02 | 0.03 | 2.16 | 5.63E-02 |
| Sex | 1 | 0.02 | 0.02 | 1.76 | 1.01E-01 |
| Type | 1 | 0.01 | 0.02 | 1.58 | 1.33E-01 |
| LPS | 1 | 0.01 | 0.01 | 0.70 | 6.21E-01 |
| Residual | 60 | 0.54 | 0.82 | NA | NA |
| Total | 67 | 0.67 | 1.00 | NA | NA |

**Supplementary Table 7.** Bacterial metabolic pathways showing association with serum Gd-IgA1 levels in IgAN cases and controls.

| **Feature** | **coef** | **stderr** | **pval** | **qval** |
| --- | --- | --- | --- | --- |
| PWY.7316..dTDP.N.acetylviosamine.biosynthesis | -1.06 | 0.45 | 1.75E-02 | 1.18E-01 |
| PWY.7332..superpathway.of.UDP.N.acetylglucosamine.derived.O.antigen.building.blocks.biosynthesis | -0.55 | 0.05 | 1.86E-26 | 4.51E-25 |
| PWY.7340..9.cis..11.trans.octadecadienoyl.CoA.degradation..isomerase.dependent..yeast. | -0.49 | 0.06 | 1.21E-15 | 2.17E-14 |
| PWY.6953..dTDP.3.acetamido..alpha..D.fucose.biosynthesis | -0.44 | 0.04 | 1.81E-26 | 4.43E-25 |
| CRNFORCAT.PWY..creatinine.degradation.I | -0.39 | 0.03 | 3.56E-29 | 9.32E-28 |
| PWY.7391..isoprene.biosynthesis.II..engineered. | -0.27 | 0.07 | 4.84E-05 | 6.13E-04 |
| PWY.6165..chorismate.biosynthesis.II..archaea. | -0.20 | 0.01 | 7.31E-118 | 4.25E-116 |
| PWY.6435..4.hydroxybenzoate.biosynthesis.III..plants. | -0.17 | 0.02 | 7.24E-17 | 1.35E-15 |
| PWY.622..starch.biosynthesis | -0.15 | 0.00 | 0.00E+00 | 0.00E+00 |
| PWY66.367..ketogenesis | -0.11 | 0.04 | 5.62E-03 | 5.30E-02 |
| PWY.7688..dTDP..alpha..D.ravidosamine.and.dTDP.4.acetyl..alpha..D.ravidosamine.biosynthesis | -0.08 | 0.02 | 1.33E-04 | 1.63E-03 |
| P621.PWY..nylon.6.oligomer.degradation | -0.06 | 0.02 | 6.19E-03 | 5.74E-02 |
| PWY.7209..superpathway.of.pyrimidine.ribonucleosides.degradation | -0.06 | 0.01 | 5.16E-18 | 9.81E-17 |
| PWY.7992..superpathway.of.menaquinol.8.biosynthesis.III | -0.05 | 0.01 | 1.11E-07 | 1.65E-06 |
| THRESYN.PWY..superpathway.of.L.threonine.biosynthesis | -0.03 | 0.01 | 2.84E-03 | 2.99E-02 |
| PWY.5695..inosine.5..phosphate.degradation | -0.03 | 0.01 | 1.84E-02 | 1.22E-01 |
| PWY.7221..guanosine.ribonucleotides.de.novo.biosynthesis | -0.03 | 0.01 | 3.30E-02 | 1.76E-01 |
| PWY.5097..L.lysine.biosynthesis.VI | -0.02 | 0.01 | 3.74E-02 | 1.91E-01 |
| PWY.724..superpathway.of.L.lysine..L.threonine.and.L.methionine.biosynthesis.II | -0.02 | 0.01 | 3.02E-02 | 1.67E-01 |
| GLUCOSE1PMETAB.PWY..glucose.and.glucose.1.phosphate.degradation | -0.02 | 0.00 | 3.32E-26 | 7.93E-25 |
| PWY.7196..superpathway.of.pyrimidine.ribonucleosides.salvage | 0.02 | 0.01 | 2.95E-03 | 3.09E-02 |
| PWY.5994..palmitate.biosynthesis..type.I.fatty.acid.synthase. | 0.03 | 0.01 | 1.85E-02 | 1.22E-01 |
| PWY.6167..flavin.biosynthesis.II..archaea. | 0.04 | 0.01 | 1.16E-03 | 1.33E-02 |
| P108.PWY..pyruvate.fermentation.to.propanoate.I | 0.04 | 0.01 | 2.99E-05 | 3.87E-04 |
| GLUCUROCAT.PWY..superpathway.of..beta..D.glucuronosides.degradation | 0.08 | 0.04 | 5.39E-02 | 2.48E-01 |
| PPGPPMET.PWY..ppGpp.metabolism | 0.09 | 0.05 | 5.42E-02 | 2.48E-01 |
| PWY.6507..4.deoxy.L.threo.hex.4.enopyranuronate.degradation | 0.09 | 0.05 | 3.79E-02 | 1.93E-01 |
| PWY.6920..6.gingerol.analog.biosynthesis..engineered. | 0.11 | 0.05 | 1.51E-02 | 1.08E-01 |
| P23.PWY..reductive.TCA.cycle.I | 0.12 | 0.01 | 3.39E-20 | 6.74E-19 |
| PWY.7094..fatty.acid.salvage | 0.13 | 0.03 | 1.10E-05 | 1.51E-04 |
| PWY.7013...S..propane.1.2.diol.degradation | 0.14 | 0.06 | 1.47E-02 | 1.05E-01 |
| PWY.6168..flavin.biosynthesis.III..fungi. | 0.15 | 0.00 | 0.00E+00 | 0.00E+00 |
| PWY.7200..superpathway.of.pyrimidine.deoxyribonucleoside.salvage | 0.15 | 0.03 | 2.44E-09 | 3.83E-08 |
| PWY.5464..superpathway.of.cytosolic.glycolysis..plants...pyruvate.dehydrogenase.and.TCA.cycle | 0.17 | 0.04 | 2.57E-05 | 3.39E-04 |
| PWY.6478..GDP.D.glycero..alpha..D.manno.heptose.biosynthesis | 0.18 | 0.04 | 2.97E-06 | 4.22E-05 |
| PWY0.1337..oleate..beta..oxidation | 0.19 | 0.03 | 1.17E-10 | 1.87E-09 |
| PWY.6415..L.ascorbate.biosynthesis.V..euglena..D.galacturonate.pathway. | 0.19 | 0.02 | 5.35E-20 | 1.05E-18 |
| PWY.6922..L.N.delta..acetylornithine.biosynthesis | 0.21 | 0.02 | 1.19E-32 | 3.57E-31 |
| PWY0.781..aspartate.superpathway | 0.22 | 0.09 | 1.85E-02 | 1.22E-01 |
| P4.PWY..superpathway.of.L.lysine..L.threonine.and.L.methionine.biosynthesis.I | 0.22 | 0.09 | 1.83E-02 | 1.22E-01 |
| PWY.7312..dTDP..beta..D.fucofuranose.biosynthesis | 0.22 | 0.02 | 3.71E-46 | 1.31E-44 |
| PWY.5845..superpathway.of.menaquinol.9.biosynthesis | 0.23 | 0.01 | 6.60E-206 | 5.06E-204 |
| PWY.5004..superpathway.of.L.citrulline.metabolism | 0.25 | 0.02 | 4.54E-48 | 1.65E-46 |
| PWY.5862..superpathway.of.demethylmenaquinol.9.biosynthesis | 0.25 | 0.01 | 3.90E-169 | 2.70E-167 |
| PWY.7315..dTDP.N.acetylthomosamine.biosynthesis | 0.27 | 0.12 | 2.08E-02 | 1.31E-01 |
| P241.PWY..coenzyme.B.biosynthesis | 0.32 | 0.04 | 1.53E-13 | 2.60E-12 |
| PWY.6285..superpathway.of.fatty.acids.biosynthesis..E..coli. | 0.32 | 0.01 | 0.00E+00 | 0.00E+00 |
| GALACTARDEG.PWY..D.galactarate.degradation.I | 0.35 | 0.17 | 3.50E-02 | 1.82E-01 |
| GLUCARGALACTSUPER.PWY..superpathway.of.D.glucarate.and.D.galactarate.degradation | 0.35 | 0.17 | 3.50E-02 | 1.82E-01 |
| PWY.6281..L.selenocysteine.biosynthesis.II..archaea.and.eukaryotes. | 0.39 | 0.07 | 4.69E-09 | 7.31E-08 |
| PWY.5392..reductive.TCA.cycle.II | 0.39 | 0.02 | 1.94E-67 | 9.07E-66 |
| PWY.6396..superpathway.of.2.3.butanediol.biosynthesis | 0.49 | 0.05 | 1.12E-26 | 2.78E-25 |
| AEROBACTINSYN.PWY..aerobactin.biosynthesis | 0.68 | 0.05 | 2.04E-48 | 7.56E-47 |
| GLYCOLYSIS.TCA.GLYOX.BYPASS..superpathway.of.glycolysis..pyruvate.dehydrogenase..TCA..and.glyoxylate.bypass | 1.34 | 0.53 | 1.12E-02 | 8.90E-02 |
| PWY.7409..phospholipid.remodeling..phosphatidylethanolamine..yeast. | 1.39 | 0.71 | 4.93E-02 | 2.33E-01 |
| PWY.7388..octanoyl..acyl.carrier.protein..biosynthesis..mitochondria..yeast. | 1.63 | 0.60 | 6.42E-03 | 5.84E-02 |
| PWY66.430..myristate.biosynthesis..mitochondria. | 1.64 | 0.60 | 6.37E-03 | 5.84E-02 |

**Supplementary Table 8.** Baseline characteristics of all IgAN patients and comparison between progressors vs. nonprogressors

| **Baseline characteristics** | **IgAN patients, n=46** | **Progressors, n = 23** | **Nonprogressors,**  **n = 23** | **p-value** |
| --- | --- | --- | --- | --- |
| Age, yr | 42 (35 - 48) | 40 (33.5 - 46) | 44 (36.5 - 54) | 0.13 |
| Male | 29 (63%) | 14 (60.9%) | 15 (65.2%) | 0.76 |
| BMI, kg/m^2^ | 26 (23.6 - 29) | 25 (22 - 27) | 26 (25 – 29.4) | 0.1 |
| Systolic BP, mmHg | 130 (124 - 145) | 131 (120 - 152) | 130 (125 - 138) | 0.52 |
| Diastolic BP, mmHg | 80 (73 - 90) | 80 (70 - 90) | 80 (75 - 83) | 0.76 |
| Serum creatinine, µmol/l | 106.5 (84 - 199) | 165 (85.5 – 248.5) | 103 (87 - 131) | 0.08 |
| eGFR, ml/min per 1.73m^2^ | 65.5 (32 - 96) | 36 (22.5 – 89.5) | 76 (52 – 97.5) | 0.048 |
| CKD stage 1 | 14 (30.4) | 6 (26.1) | 8 (34.8) |  |
| CKD stage 2 | 10 (21.7) | 4 (17.4) | 6 (26.1) |  |
| CKD stage 3 | 11 (23.9) | 4 (17.4) | 7 (30.4) |  |
| CKD stage 4 | 9 (19.6) | 7 (30.4) | 2 (8.7) |  |
| CKD stage 5 without dialysis | 2 (4.3) | 2 (8.7) | 0 |  |
| UPCR, g/g | 0.4 (0.1 – 0.9) | 0.62 (0.4 – 1.55) | 0.15 (0.09 – 0.39) | 0.002 |
| Hematuria, RBC/µl | 20 (6 – 57.6) | 22.1 (8.6 – 96.25) | 10.8 (2.75 – 47.4) | 0.12 |
| Serum total cholesterol, mmol/l | 5.26 (4.78 – 5.81) | 5.26 (4.78 – 5.6) | 5.26 (4.76 – 5.86) | 0.85 |
| Gd-IgA1, ng/ml | 6809 (4986 - 9414) | 5354 (4093.5 - 8179) | 8175 (6175 - 10465) | 0.03 |
| Lipopolysaccharides, pg/ml | 139.3 (107.7 – 190.9) | 128.4 (106.2 – 181.95) | 150.3 (124 – 195.6) | 0.52 |
| Indoxyl sulfate, ng/ml | 243.8 (199.6 – 377.2) | 250.7 (187.75 - 391) | 243.7 (212.6 – 302.6) | 0.89 |
| Mesangial hypercellularity (M1) | 34 (73.9) | 17 (73.9) | 17 (73.9) | 1.0 |
| Endocapillary hypercellularity (E1) | 2 (4.3) | 0 | 2 (8.7) | 0.15 |
| Segmental glomerulosclerosis (S1) | 31 (67.4) | 14 (60.9) | 17 (73.9) | 0.35 |
| Tubular atrophy/interstitial fibrosis 26–50% (T1) | 3 (6.5) | 3 (13) | 0 | 0.2 |
| Tubular atrophy/interstitial fibrosis >50% (T2) | 2 (4.3) | 1 (4.3) | 1 (4.3) | 1.0 |
| Cellular/fibrocellular crescents <25% (C1) | 3 (6.5) | 1 (4.3) | 2 (8.7) | 0.55 |

The results are expressed as the median (interquartile range) or n (%).

**Supplementary Table 9.** Comparison of various alpha diversity measures between IgAN nonprogressors and progressors (Wilcoxon test, Benjamini-Hochberg procedure).

| **Method** | **Nonprogressors (median, IQR)** | **Progressors (median, IQR)** | **p** | **p.adj** |
| --- | --- | --- | --- | --- |
| Observed taxa | 2046.00 (1956.00 - 2097.00) | 2043.00 (1862.00 - 2133.00) | 0.74 | 7.40E-01 |
| Pielou's evenness | 0.65 (0.63 - 0.68) | 0.65 (0.62 - 0.68) | 0.36 | 3.60E-01 |
| Shannon index | 4.92 (4.82 - 5.24) | 4.95 (4.69 - 5.19) | 0.36 | 3.60E-01 |
| Inverse Simpson index | 61.85 (51.14 - 86.54) | 53.86 (35.98 - 75.85) | 0.2 | 2.00E-01 |
| Chao1 | 2046.00 (1956.00 - 2097.00) | 2043.00 (1862.00 - 2133.00) | 0.74 | 7.40E-01 |


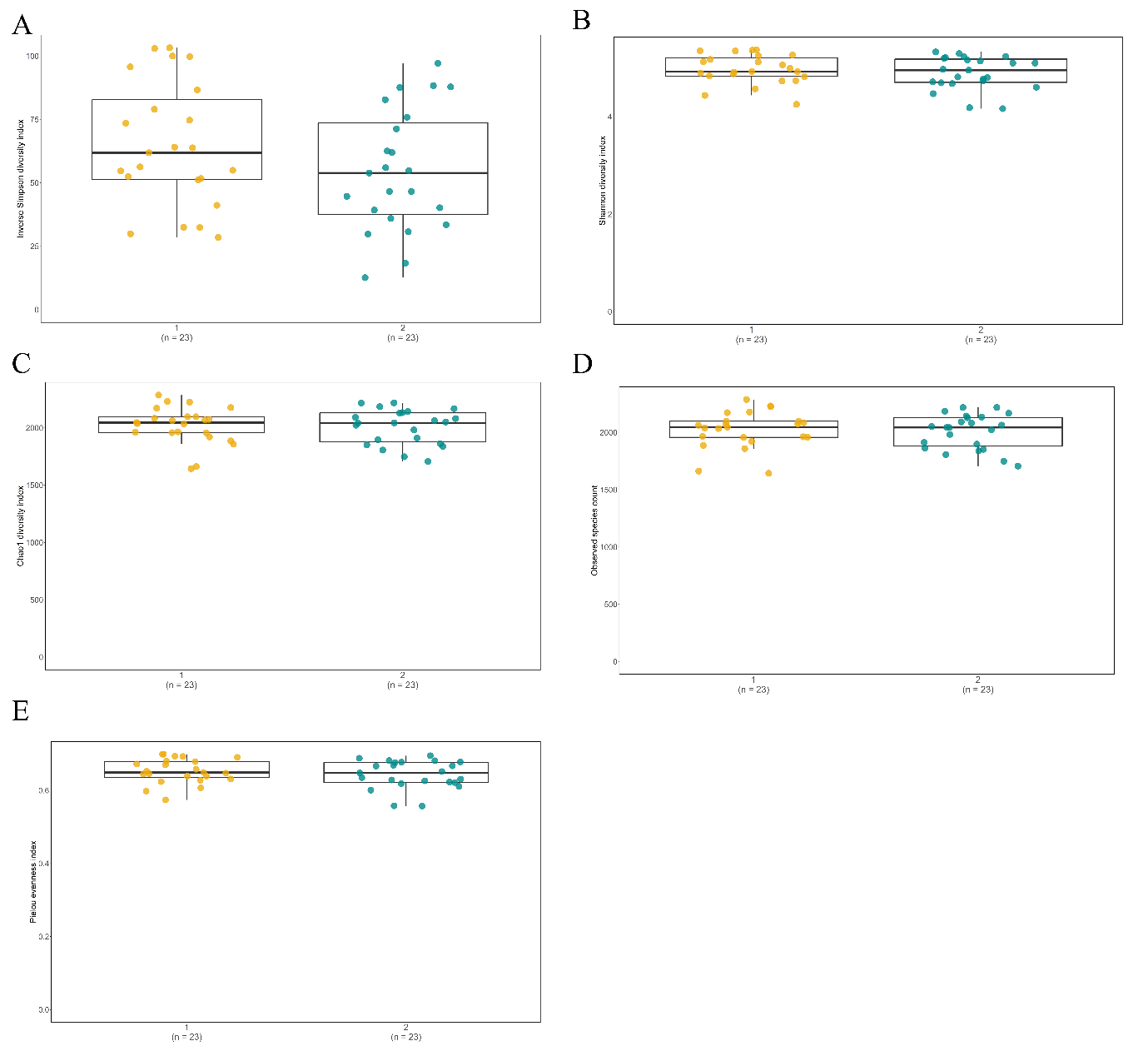


**Supplementary Figure 3. Alpha diversity measures of IgA nephropathy nonprogressors and progressors:** A – Inverse Simpson diversity index; B – Shannon diversity index; C – Chao1 diversity index; D – Observed taxa; E – Pielou's evenness index. Boxplots present the median, 25th, and 75th percentiles. 1 – nonprogressors; 2 – progressors.

**Supplementary Table 10.** Contributing factors determining the inter-sample variation of gut microbiome profiles in IgA nephropathy patients only identified by PERMANOVA.

| **Variable** | **Df** | **Sum Of Sqs** | **R2** | **F** | **Pr (>F)** |
| --- | --- | --- | --- | --- | --- |
| Progression | 1 | 0.31 | 0.03 | 1.43 | 3.97E-02 |
| BMI | 1 | 0.28 | 0.03 | 1.31 | 7.95E-02 |
| Hematuria | 1 | 0.24 | 0.02 | 1.11 | 2.59E-01 |
| LPS | 1 | 0.23 | 0.02 | 1.09 | 2.88E-01 |
| eGFR | 1 | 0.22 | 0.02 | 1.04 | 3.61E-01 |
| Sex | 1 | 0.22 | 0.02 | 1.01 | 4.11E-01 |
| Indoxylsulfate | 1 | 0.21 | 0.02 | 0.98 | 4.92E-01 |
| Proteinuria range | 1 | 0.20 | 0.02 | 0.96 | 5.30E-01 |
| Gd-IgA1 | 1 | 0.19 | 0.02 | 0.91 | 6.34E-01 |
| Prognosis | 1 | 0.16 | 0.02 | 0.74 | 9.33E-01 |
| Residual | 35 | 7.48 | 0.77 | NA | NA |
| Total | 45 | 9.73 | 1.00 | NA | NA |


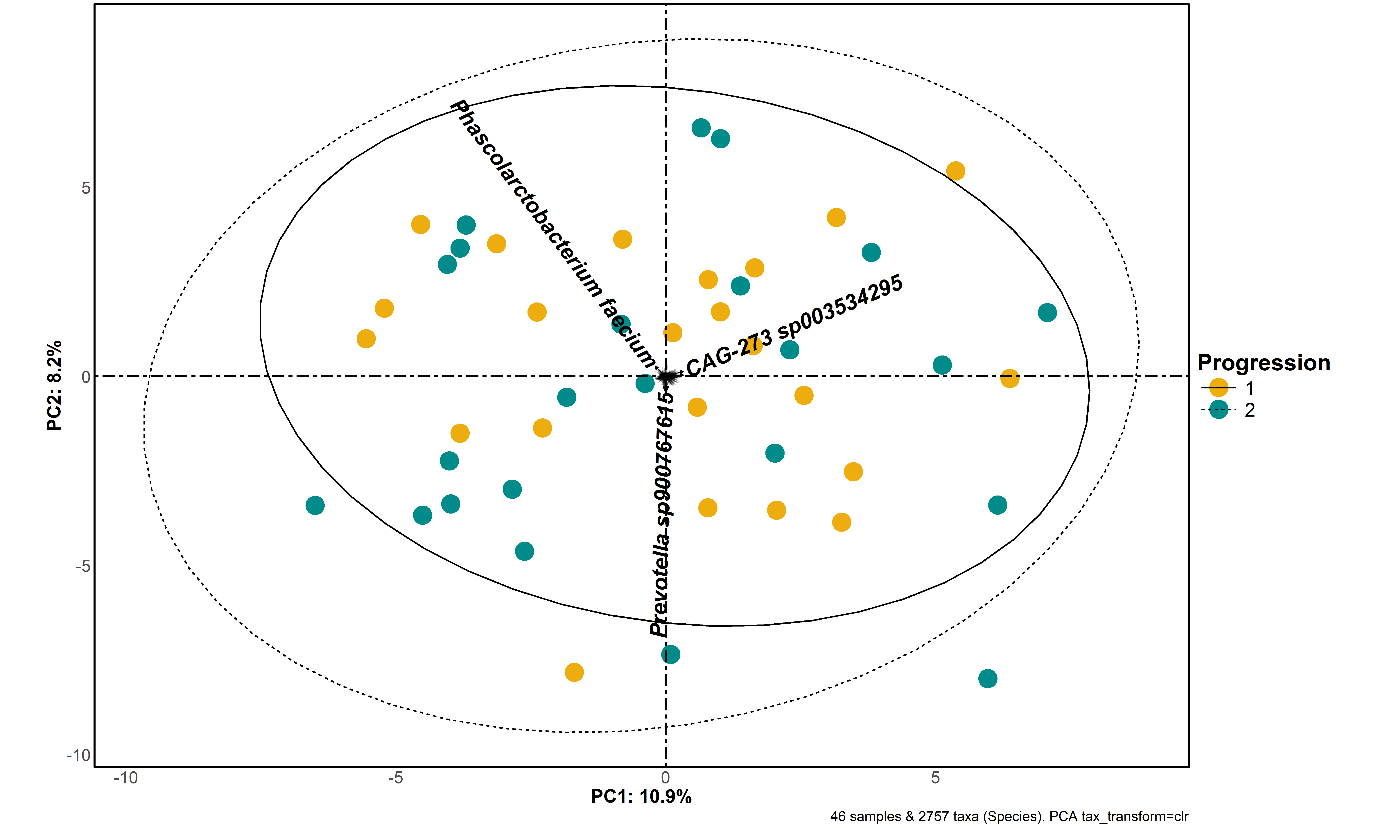


**Supplementary Figure 4.** Principal component analysis plot showing inter-sample variability in microbial community composition among IgA nephropathy nonprogressors (1) and progressors (2).

**Supplementary Table 11.** The absolute and relative abundance of the gut microbiome taxa in IgA nephropathy progressors and nonprogressors.

| **Species** | **Class** | **Progression** | **Abundance by progression** | **Relative abundance by progression** |
| --- | --- | --- | --- | --- |
| *s__Prevotella sp900557255* | Species | progressors | 19776645 | 4.4 |
| *s__Prevotella sp900557255* | Species | nonprogressors | 6751674 | 1.45 |
| *s__Fusicatenibacter saccharivorans* | Species | progressors | 12738158 | 2.83 |
| *s__Fusicatenibacter saccharivorans* | Species | nonprogressors | 10157639 | 2.18 |
| *s__Phocaeicola dorei* | Species | progressors | 12923605 | 2.87 |
| *s__Phocaeicola dorei* | Species | nonprogressors | 9398084 | 2.02 |
| *s__Agathobacter rectalis* | Species | progressors | 7981042 | 1.77 |
| *s__Agathobacter rectalis* | Species | nonprogressors | 12449323 | 2.67 |
| *s__Blautia_A wexlerae_A* | Species | progressors | 8863173 | 1.97 |
| *s__Blautia_A wexlerae_A* | Species | nonprogressors | 9292359 | 2 |
| *s__Bacteroides uniformis* | Species | progressors | 8147439 | 1.81 |
| *s__Bacteroides uniformis* | Species | nonprogressors | 7326545 | 1.57 |
| *s__Bifidobacterium adolescentis* | Species | progressors | 5115567 | 1.14 |
| *s__Bifidobacterium adolescentis* | Species | nonprogressors | 6685058 | 1.44 |
| *s__Faecalibacterium prausnitzii_C* | Species | progressors | 5982185 | 1.33 |
| *s__Faecalibacterium prausnitzii_C* | Species | nonprogressors | 5529239 | 1.19 |
| *s__Agathobacter faecis* | Species | progressors | 4767276 | 1.06 |
| *s__Agathobacter faecis* | Species | nonprogressors | 5396476 | 1.16 |
| *s__Ruminococcus_E bromii_B* | Species | progressors | 5665962 | 1.26 |
| *s__Ruminococcus_E bromii_B* | Species | nonprogressors | 4142082 | 0.89 |
| *s__Faecalibacterium prausnitzii_G* | Species | progressors | 3489914 | 0.78 |
| *s__Faecalibacterium prausnitzii_G* | Species | nonprogressors | 6068322 | 1.3 |
| *s__Anaerostipes hadrus* | Species | progressors | 3730590 | 0.83 |
| *s__Anaerostipes hadrus* | Species | nonprogressors | 5435106 | 1.17 |
| *s__Gemmiger qucibialis* | Species | progressors | 3706465 | 0.82 |
| *s__Gemmiger qucibialis* | Species | nonprogressors | 5121174 | 1.1 |
| *s__UBA11524 sp000437595* | Species | progressors | 4101926 | 0.91 |
| *s__UBA11524 sp000437595* | Species | nonprogressors | 3807623 | 0.82 |
| *s__Alistipes putredinis* | Species | progressors | 3250661 | 0.72 |
| *s__Alistipes putredinis* | Species | nonprogressors | 4247297 | 0.91 |
| *s__Dorea_A longicatena* | Species | progressors | 4467321 | 0.99 |
| *s__Dorea_A longicatena* | Species | nonprogressors | 2983893 | 0.64 |
| *s__Mediterraneibacter faecis* | Species | progressors | 3790277 | 0.84 |
| *s__Mediterraneibacter faecis* | Species | nonprogressors | 3411035 | 0.73 |
| *s__Blautia_A massiliensis* | Species | progressors | 3521718 | 0.78 |
| *s__Blautia_A massiliensis* | Species | nonprogressors | 3555120 | 0.76 |
| *s__Prevotella sp900546535* | Species | progressors | 2138586 | 0.48 |
| *s__Prevotella sp900546535* | Species | nonprogressors | 4932272 | 1.06 |
| *s__Ruminococcus_D bicirculans* | Species | progressors | 3584087 | 0.8 |
| *s__Ruminococcus_D bicirculans* | Species | nonprogressors | 3353999 | 0.72 |
| *Others* | Species | progressors | 322026658 | 71.6 |
| *Others* | Species | nonprogressors | 345562039 | 74.22 |

**Supplementary Table 12.** The differential abundance analysis at the species level comparing the gut microbiome composition of progressors versus non-progressors.

| **Feature** | **coef** | **stderr** | **pval** | **qval** |
| --- | --- | --- | --- | --- |
| CAG.1427.sp000434775 | -11.24 | 0.01 | 0 | 0 |
| CAG.1427.sp000436075 | -8.39 | 0.00 | 0 | 0 |
| CAG.267.sp900551865 | -7.04 | 0.05 | 0 | 0 |
| CAG.302.sp900549325 | -6.50 | 0.02 | 0 | 0 |
| CAG.312.sp000438015 | -5.94 | 0.01 | 0 | 0 |
| CAG.312.sp900545715 | -5.90 | 0.04 | 0 | 0 |
| CAG.533.sp900553855 | -5.72 | 0.01 | 0 | 0 |
| CAG.611.sp000434175 | -5.69 | 0.01 | 0 | 0 |
| CAG.611.sp900552235 | -5.66 | 0.02 | 0 | 0 |
| CAG.698.sp000431235 | -5.29 | 0.05 | 0 | 0 |
| Catenibacterium.sp900764565 | -5.24 | 0.01 | 0 | 0 |
| Citrobacter.freundii | -5.21 | 0.04 | 0 | 0 |
| Collinsella.aerofaciens_A | -5.17 | 0.03 | 0 | 0 |
| Collinsella.aerofaciens_K | -5.00 | 0.02 | 0 | 0 |
| Collinsella.sp003439125 | -4.74 | 0.02 | 0 | 0 |
| Collinsella.sp003469185 | -4.65 | 0.01 | 0 | 0 |
| Collinsella.sp900541035 | -4.55 | 0.03 | 0 | 0 |
| Collinsella.sp900541065 | -4.48 | 0.01 | 0 | 0 |
| Collinsella.sp900541135 | -4.48 | 0.02 | 0 | 0 |
| Collinsella.sp900541645 | -4.45 | 0.03 | 0 | 0 |
| Collinsella.sp900542125 | -4.44 | 0.02 | 0 | 0 |
| Collinsella.sp900542165 | -4.39 | 0.02 | 0 | 0 |
| Collinsella.sp900542305 | -4.33 | 0.03 | 0 | 0 |
| Collinsella.sp900542315 | -4.17 | 0.02 | 0 | 0 |
| Collinsella.sp900542325 | -3.89 | 0.02 | 0 | 0 |
| Collinsella.sp900544135 | -3.88 | 0.01 | 0 | 0 |
| Collinsella.sp900544205 | -3.76 | 0.02 | 0 | 0 |
| Collinsella.sp900544845 | -3.76 | 0.01 | 0 | 0 |
| Collinsella.sp900545615 | -3.76 | 0.04 | 0 | 0 |
| Collinsella.sp900547805 | -3.75 | 0.04 | 0 | 0 |
| Collinsella.sp900549345 | -3.72 | 0.02 | 0 | 0 |
| Collinsella.sp900549555 | -3.71 | 0.02 | 0 | 0 |
| Collinsella.sp900551195 | -3.67 | 0.01 | 0 | 0 |
| Collinsella.sp900551555 | -3.66 | 0.01 | 0 | 0 |
| Collinsella.sp900756725 | -3.56 | 0.01 | 0 | 0 |
| Collinsella.sp900758375 | -3.51 | 0.01 | 0 | 0 |
| Collinsella.sp900761945 | -3.51 | 0.02 | 0 | 0 |
| Collinsella.sp900762345 | -3.50 | 0.03 | 0 | 0 |
| Desulfovibrio.piger | -3.49 | 0.00 | 0 | 0 |
| Desulfovibrio.piger_A | -3.48 | 0.00 | 0 | 0 |
| Duodenibacillus.sp900762555 | -3.47 | 0.03 | 0 | 0 |
| Holdemanella.sp003436425 | -3.42 | 0.00 | 0 | 0 |
| Klebsiella_A.oxytoca | -3.41 | 0.04 | 0 | 0 |
| Kluyvera.ascorbata_B | -3.39 | 0.06 | 0 | 0 |
| Lactobacillus.acidophilus | -3.38 | 0.02 | 0 | 0 |
| Mailhella.sp900541395 | -3.34 | 0.01 | 0 | 0 |
| Megasphaera.sp000417505 | -3.34 | 0.02 | 0 | 0 |
| Methanosphaera.stadtmanae | -3.34 | 0.01 | 0 | 0 |
| Mitsuokella.jalaludinii | -3.31 | 0.01 | 0 | 0 |
| Mitsuokella.multacida | -3.31 | 0.03 | 0 | 0 |
| Mitsuokella.sp003458855 | -3.29 | 0.10 | 0 | 0 |
| Mitsuokella.sp900552565 | -3.28 | 0.05 | 0 | 0 |
| Phascolarctobacterium.faecium | -3.27 | 0.00 | 0 | 0 |
| Phascolarctobacterium.sp900551745 | -3.20 | 0.01 | 0 | 0 |
| Phascolarctobacterium_A.sp900770955 | -3.18 | 0.00 | 0 | 0 |
| Senegalimassilia.anaerobia | -3.16 | 0.00 | 0 | 0 |
| Succinivibrio.sp000431835 | -3.12 | 0.08 | 0 | 0 |
| Sutterella.faecalis | -3.11 | 0.01 | 0 | 0 |
| UBA10677.sp900553005 | -3.10 | 0.02 | 0 | 0 |
| UBA733.sp900550795 | -3.09 | 0.06 | 0 | 0 |
| UMGS1124.sp900548725 | -3.08 | 0.05 | 0 | 0 |
| UMGS1293.sp900754495 | -3.07 | 0.01 | 0 | 0 |
| UMGS1477.sp900552205 | -3.05 | 0.03 | 0 | 0 |
| UMGS1477.sp900761525 | -3.05 | 0.03 | 0 | 0 |
| UMGS1688.sp900554085 | -3.04 | 0.02 | 0 | 0 |
| UMGS687.sp900544595 | -3.03 | 0.06 | 0 | 0 |
| UMGS687.sp900551705 | -2.98 | 0.11 | 0 | 0 |
| Varibaculum.timonense | -2.91 | 0.02 | 0 | 0 |
| Weissella.cibaria | -2.90 | 0.03 | 0 | 0 |
| Zag111.sp002102825 | -2.89 | 0.01 | 0 | 0 |
| Selenomonas_C.bovis | -2.89 | 0.10 | 2.24E-303 | 9.58E-302 |
| Collinsella.sp900546115 | -2.88 | 0.05 | 1.06E-302 | 4.52E-301 |
| Collinsella.sp900552705 | -2.85 | 0.02 | 5.55E-300 | 2.34E-298 |
| Collinsella.sp900542635 | -2.85 | 0.01 | 5.18E-294 | 2.16E-292 |
| Collinsella.sp900761615 | -2.82 | 0.02 | 3.80E-261 | 1.54E-259 |
| Collinsella.sp900757385 | -2.75 | 0.04 | 1.23E-251 | 4.95E-250 |
| Collinsella.sp003470665 | -2.74 | 0.02 | 5.21E-240 | 2.08E-238 |
| Collinsella.sp900544095 | -2.71 | 0.02 | 1.56E-229 | 6.11E-228 |
| UMGS1670.sp900553995 | -2.69 | 0.01 | 6.45E-222 | 2.49E-220 |
| RUG705.sp900551455 | -2.66 | 0.04 | 1.41E-218 | 5.38E-217 |
| Enterobacter.kobei | -2.64 | 0.10 | 1.76E-211 | 6.63E-210 |
| Collinsella.sp900542945 | -2.60 | 0.08 | 1.36E-204 | 4.99E-203 |
| Collinsella.sp900557455 | -2.58 | 0.02 | 7.65E-204 | 2.79E-202 |
| Collinsella.sp900549335 | -2.53 | 0.02 | 2.61E-195 | 9.47E-194 |
| Limosilactobacillus.sp012843675 | -2.51 | 0.05 | 2.58E-188 | 9.29E-187 |
| Hafnia.paralvei | -2.50 | 0.02 | 4.90E-188 | 1.75E-186 |
| QAMH01.sp900544245 | -2.48 | 0.00 | 1.96E-182 | 6.90E-181 |
| QANA01.sp900554725 | -2.48 | 0.01 | 1.07E-181 | 3.74E-180 |
| CAG.485.sp002361235 | -2.48 | 0.10 | 1.24E-179 | 4.30E-178 |
| CAG.977.sp000434295 | -2.47 | 0.04 | 1.43E-175 | 4.88E-174 |
| Hafnia.alvei | -2.45 | 0.13 | 1.19E-161 | 4.00E-160 |
| Anaeroglobus.massiliensis | -2.44 | 0.06 | 3.98E-155 | 1.30E-153 |
| Prevotella.buccae | -2.40 | 0.12 | 9.26E-155 | 3.01E-153 |
| Enorma.timonensis | -2.38 | 0.05 | 2.05E-154 | 6.61E-153 |
| Collinsella.sp900544995 | -2.37 | 0.02 | 5.76E-152 | 1.84E-150 |
| UBA4372.sp900766785 | -2.37 | 0.12 | 1.47E-147 | 4.65E-146 |
| UBA4877.sp002399345 | -2.37 | 0.02 | 4.43E-146 | 1.40E-144 |
| Varibaculum.cambriense_A | -2.36 | 0.06 | 5.12E-141 | 1.61E-139 |
| CAG.631.sp000433015 | -2.35 | 0.07 | 1.42E-140 | 4.44E-139 |
| Cronobacter.malonaticus | -2.34 | 0.07 | 3.17E-140 | 9.84E-139 |
| Eisenbergiella.sp900548905 | -2.33 | 0.09 | 3.42E-140 | 1.05E-138 |
| Collinsella.sp900761995 | -2.32 | 0.03 | 3.00E-136 | 9.14E-135 |
| Collinsella.sp900754275 | -2.30 | 0.03 | 5.16E-136 | 1.55E-134 |
| Collinsella.sp900546105 | -2.29 | 0.06 | 1.44E-133 | 4.29E-132 |
| Enterococcus_D.sp002850555 | -2.29 | 0.10 | 9.35E-133 | 2.76E-131 |
| Collinsella.sp900761035 | -2.29 | 0.03 | 7.95E-131 | 2.32E-129 |
| Collinsella.sp900766165 | -2.29 | 0.03 | 7.63E-128 | 2.19E-126 |
| Anaeroglobus.geminatus | -2.28 | 0.04 | 3.57E-124 | 1.00E-122 |
| Caecibacter.massiliensis | -2.23 | 0.05 | 4.28E-122 | 1.20E-120 |
| Collinsella.sp900540095 | -2.22 | 0.03 | 1.89E-117 | 5.26E-116 |
| Parascardovia.denticolens | -2.21 | 0.08 | 3.40E-116 | 9.42E-115 |
| Collinsella.sp003466125 | -2.20 | 0.03 | 5.72E-111 | 1.53E-109 |
| CAG.826.sp000437235 | -2.19 | 0.01 | 1.72E-109 | 4.57E-108 |
| Duodenibacillus.sp900544335 | -2.18 | 0.01 | 2.22E-107 | 5.87E-106 |
| Phascolarctobacterium.sp900545535 | -2.17 | 0.02 | 5.83E-105 | 1.53E-103 |
| Anaeroplasma.sp900767915 | -2.16 | 0.06 | 3.51E-99 | 9.11E-98 |
| Collinsella.sp900540905 | -2.15 | 0.03 | 7.00E-99 | 1.81E-97 |
| Collinsella.sp900754445 | -2.14 | 0.02 | 6.95E-95 | 1.74E-93 |
| UMGS1601.sp900553335 | -2.14 | 0.07 | 2.10E-91 | 5.24E-90 |
| Acidaminococcus.sp900538365 | -2.12 | 0.08 | 2.74E-90 | 6.78E-89 |
| Zag111.sp003258735 | -2.11 | 0.06 | 3.43E-90 | 8.45E-89 |
| Collinsella.sp900545995 | -2.11 | 0.04 | 9.49E-89 | 2.30E-87 |
| Sodaliphilus.pleomorphus | -2.06 | 0.16 | 1.02E-87 | 2.44E-86 |
| Olsenella_B.sp900768455 | -2.06 | 0.07 | 1.98E-82 | 4.67E-81 |
| Oxalobacter.formigenes_B | -2.05 | 0.06 | 6.67E-80 | 1.53E-78 |
| UBA7173.sp001701135 | -2.05 | 0.20 | 2.90E-79 | 6.60E-78 |
| Enterobacter_D.kobei_A | -2.05 | 0.05 | 8.77E-79 | 1.99E-77 |
| Anaeroglobus.micronuciformis | -2.04 | 0.10 | 1.46E-77 | 3.26E-76 |
| CAG.115.sp900766795 | -2.04 | 0.05 | 3.69E-76 | 8.20E-75 |
| UBA1174.sp900556855 | -2.04 | 0.05 | 2.17E-73 | 4.78E-72 |
| Campylobacter_A.concisus_R | -2.01 | 0.06 | 4.78E-73 | 1.05E-71 |
| Lactococcus.lactis_E | 2.00 | 0.26 | 5.32E-02 | 1.64E-01 |
| CAG.495.sp900540425 | 2.00 | 0.51 | 5.35E-02 | 1.64E-01 |
| Frisingicoccus.caecimuris | 2.00 | 0.15 | 5.36E-02 | 1.64E-01 |
| UMGS1241.sp900540495 | 2.03 | 0.24 | 5.36E-02 | 1.64E-01 |
| HGM11514.sp900757255 | 2.04 | 0.22 | 5.36E-02 | 1.64E-01 |
| Parascardovia.denticolens | 2.06 | 0.03 | 5.38E-02 | 1.64E-01 |
| Anaerostipes.hadrus_A | 2.06 | 0.17 | 5.38E-02 | 1.65E-01 |
| Blautia_A.obeum_B | 2.07 | 0.13 | 5.40E-02 | 1.65E-01 |
| Phil1.sp001940855 | 2.07 | 0.27 | 5.41E-02 | 1.65E-01 |
| Agathobaculum.sp900555465 | 2.08 | 0.09 | 5.45E-02 | 1.66E-01 |
| Methanobrevibacter_A.woesei | 2.09 | 0.29 | 5.46E-02 | 1.66E-01 |
| UBA5416.sp900539175 | 2.09 | 0.23 | 5.46E-02 | 1.66E-01 |
| X1XD42.69.sp014287635 | 2.09 | 0.20 | 5.48E-02 | 1.67E-01 |
| UBA11517.sp900555365 | 2.10 | 0.14 | 5.56E-02 | 1.69E-01 |
| CAG.557.sp000435275 | 2.10 | 0.36 | 5.60E-02 | 1.70E-01 |
| Dysosmobacter.sp900763685 | 2.12 | 0.21 | 5.60E-02 | 1.70E-01 |
| UMGS902.sp003343845 | 2.12 | 0.32 | 5.62E-02 | 1.70E-01 |
| Blautia_A.sp900120195 | 2.13 | 0.13 | 5.64E-02 | 1.70E-01 |
| Sodaliphilus.sp004557565 | 2.15 | 0.42 | 5.64E-02 | 1.70E-01 |
| HGM20899.sp900767005 | 2.17 | 0.84 | 5.65E-02 | 1.71E-01 |
| Phocaeicola.mediterraneensis | 2.18 | 0.15 | 5.68E-02 | 1.71E-01 |
| Rothia.mucilaginosa_A | 2.18 | 0.47 | 5.68E-02 | 1.71E-01 |
| Negativibacillus.sp000435195 | 2.18 | 0.40 | 5.69E-02 | 1.71E-01 |
| Lentilactobacillus.buchneri | 2.19 | 0.52 | 5.73E-02 | 1.72E-01 |
| UMGS1820.sp900555375 | 2.20 | 0.43 | 5.73E-02 | 1.72E-01 |
| CAG.510.sp900550475 | 2.20 | 0.28 | 5.74E-02 | 1.72E-01 |
| UBA11512.sp003522145 | 2.23 | 0.37 | 5.79E-02 | 1.74E-01 |
| CAG.448.sp003150135 | 2.23 | 0.26 | 5.85E-02 | 1.75E-01 |
| Collinsella.sp900540985 | 2.25 | 0.13 | 5.85E-02 | 1.75E-01 |
| Collinsella.sp900754445 | 2.25 | 0.01 | 5.85E-02 | 1.75E-01 |
| Emergencia.sp900066695 | 2.26 | 0.12 | 5.85E-02 | 1.75E-01 |
| CAG.269.sp900553125 | 2.28 | 0.28 | 5.88E-02 | 1.75E-01 |
| UBA1394.sp900066845 | 2.28 | 0.37 | 5.90E-02 | 1.76E-01 |
| Bacteroides.faecis | 2.29 | 0.25 | 5.92E-02 | 1.76E-01 |
| Clostridium.paraputrificum | 2.32 | 0.54 | 5.92E-02 | 1.76E-01 |
| UMGS1858.sp900555705 | 2.33 | 0.06 | 5.97E-02 | 1.77E-01 |
| Sellimonas.intestinalis | 2.34 | 0.17 | 5.99E-02 | 1.77E-01 |
| Oxalobacter.sp900760095 | 2.34 | 0.35 | 6.01E-02 | 1.78E-01 |
| CAG.110.sp900546915 | 2.41 | 0.27 | 6.06E-02 | 1.79E-01 |
| Lachnospira.rogosae_A | 2.42 | 0.19 | 6.07E-02 | 1.79E-01 |
| CAG.269.sp900555615 | 2.43 | 0.22 | 6.18E-02 | 1.81E-01 |
| Prevotella.sp900553465 | 2.44 | 0.26 | 6.17E-02 | 1.81E-01 |
| Gemmiger.sp900540595 | 2.45 | 0.14 | 6.21E-02 | 1.82E-01 |
| UMGS1002.sp900547565 | 2.45 | 0.09 | 6.24E-02 | 1.82E-01 |
| Alistipes.senegalensis | 2.45 | 0.17 | 6.26E-02 | 1.83E-01 |
| Campylobacter_A.concisus_F | 2.47 | 0.04 | 6.27E-02 | 1.83E-01 |
| Faecalicoccus.sp900546545 | 2.49 | 0.20 | 6.29E-02 | 1.83E-01 |
| Marvinbryantia.sp900550755 | 2.50 | 0.14 | 6.28E-02 | 1.83E-01 |
| Citrobacter.youngae | 2.50 | 0.72 | 6.36E-02 | 1.85E-01 |
| Stoquefichus.massiliensis | 2.51 | 0.87 | 6.40E-02 | 1.86E-01 |
| Collinsella.sp900752015 | 2.51 | 0.21 | 6.41E-02 | 1.86E-01 |
| CAG.196.sp900549855 | 2.51 | 0.14 | 6.42E-02 | 1.86E-01 |
| Monoglobus.pectinilyticus | 2.54 | 0.22 | 6.47E-02 | 1.87E-01 |
| CAG.303.sp900539455 | 2.54 | 0.21 | 6.49E-02 | 1.88E-01 |
| UMGS1484.sp902388225 | 2.58 | 0.42 | 6.56E-02 | 1.89E-01 |
| UBA11475.sp003538975 | 2.59 | 0.39 | 6.69E-02 | 1.92E-01 |
| UMGS1976.sp900556765 | 2.59 | 0.22 | 6.74E-02 | 1.94E-01 |
| CAG.724.sp900551005 | 2.62 | 0.14 | 6.76E-02 | 1.94E-01 |
| Clostridium.cuniculi | 2.66 | 0.24 | 6.76E-02 | 1.94E-01 |
| Pauljensenia.bouchesdurhonensis | 2.67 | 0.26 | 6.77E-02 | 1.94E-01 |
| Collinsella.sp900540905 | 2.68 | 0.01 | 6.78E-02 | 1.94E-01 |
| Roseburia.sp900550935 | 2.68 | 0.26 | 6.83E-02 | 1.96E-01 |
| Anaerostipes.caccae | 2.69 | 0.46 | 6.85E-02 | 1.96E-01 |
| Prevotella.sp000436035 | 2.69 | 0.32 | 6.96E-02 | 1.99E-01 |
| Bulleidia.massiliensis_B | 2.69 | 0.09 | 6.97E-02 | 1.99E-01 |
| UMGS1490.sp900547395 | 2.73 | 0.76 | 6.97E-02 | 1.99E-01 |
| CAG.127.sp900553925 | 2.73 | 0.23 | 7.07E-02 | 2.01E-01 |
| Coprococcus_A.sp900548825 | 2.74 | 0.13 | 7.05E-02 | 2.01E-01 |
| Enterobacter.roggenkampii | 2.77 | 1.00 | 7.07E-02 | 2.01E-01 |
| UBA11774.sp003507655 | 2.79 | 0.19 | 7.07E-02 | 2.01E-01 |
| Victivallis.sp900551245 | 2.80 | 0.45 | 7.07E-02 | 2.01E-01 |
| CAG.521.sp900553105 | 2.80 | 1.69 | 7.10E-02 | 2.01E-01 |
| Bacteroides.gallinarum | 2.81 | 0.15 | 7.12E-02 | 2.02E-01 |
| Blautia.hansenii | 2.81 | 0.19 | 7.12E-02 | 2.02E-01 |
| Prevotella.sp000436595 | 2.82 | 0.14 | 7.12E-02 | 2.02E-01 |
| Blautia.sp900547685 | 2.82 | 0.13 | 7.18E-02 | 2.03E-01 |
| GCA.900066755.sp900066755 | 2.83 | 0.13 | 7.24E-02 | 2.04E-01 |
| CAG.449.sp900551385 | 2.87 | 0.38 | 7.28E-02 | 2.06E-01 |
| Clostridium.sp900766315 | 2.88 | 0.31 | 7.33E-02 | 2.07E-01 |
| Ruminococcus_D.sp900543145 | 2.92 | 0.27 | 7.34E-02 | 2.07E-01 |
| Faecalimonas.sp900546325 | 2.94 | 0.13 | 7.35E-02 | 2.07E-01 |
| UMGS1623.sp900553525 | 3.02 | 0.17 | 7.35E-02 | 2.07E-01 |
| Bacteroides.cellulosilyticus | 3.13 | 0.23 | 7.41E-02 | 2.08E-01 |
| HGM11327.sp900759935 | 3.13 | 0.26 | 7.43E-02 | 2.09E-01 |
| Collinsella.sp000434535 | 3.14 | 0.18 | 7.46E-02 | 2.09E-01 |
| Blautia.sp900555025 | 3.14 | 0.14 | 7.49E-02 | 2.10E-01 |
| UBA737.sp900543215 | 3.23 | 0.20 | 7.53E-02 | 2.11E-01 |
| UMGS973.sp900547295 | 3.26 | 0.26 | 7.61E-02 | 2.13E-01 |
| Bacillus_A.luti | 3.28 | 0.33 | 7.63E-02 | 2.13E-01 |
| CAG.312.sp900760055 | 3.34 | 0.39 | 7.64E-02 | 2.13E-01 |
| Ruminococcus_C.sp000980705 | 3.34 | 0.36 | 7.65E-02 | 2.14E-01 |
| Faecalibacterium.prausnitzii_C | 3.38 | 0.13 | 7.69E-02 | 2.14E-01 |
| Gemella.sp900555985 | 3.39 | 0.04 | 7.72E-02 | 2.15E-01 |
| Streptococcus.sp900550895 | 3.43 | 0.18 | 7.75E-02 | 2.15E-01 |
| UBA1777.sp900549645 | 3.43 | 0.19 | 7.75E-02 | 2.15E-01 |
| CAG.95.sp000438155 | 3.52 | 0.20 | 7.76E-02 | 2.16E-01 |
| UBA1777.sp900546515 | 3.57 | 0.11 | 7.82E-02 | 2.17E-01 |
| Blautia.sp001304935 | 3.57 | 0.14 | 7.84E-02 | 2.17E-01 |
| RC9.sp900771005 | 3.63 | 0.14 | 7.84E-02 | 2.17E-01 |
| UMGS1370.sp900542035 | 3.65 | 0.14 | 7.86E-02 | 2.18E-01 |
| Agathobaculum.butyriciproducens | 3.66 | 0.09 | 7.99E-02 | 2.21E-01 |
| UCG.010.sp003150215 | 3.68 | 0.41 | 8.00E-02 | 2.21E-01 |
| Parabacteroides.johnsonii | 3.71 | 0.17 | 8.08E-02 | 2.22E-01 |
| UMGS75.sp900538885 | 3.74 | 0.32 | 8.10E-02 | 2.22E-01 |
| Vagococcus.sp003462485 | 3.75 | 0.07 | 8.10E-02 | 2.22E-01 |
| UBA1777.sp003150355 | 3.85 | 0.27 | 8.13E-02 | 2.23E-01 |
| Angelakisella.sp003453215 | 3.85 | 0.16 | 8.16E-02 | 2.24E-01 |
| CAG.433.sp000433675 | 3.86 | 0.50 | 8.21E-02 | 2.25E-01 |
| CAG.127.sp900767585 | 3.89 | 0.17 | 8.24E-02 | 2.25E-01 |
| UMGS1600.sp900553315 | 3.90 | 0.16 | 8.26E-02 | 2.26E-01 |
| Clostridium.disporicum_A | 3.98 | 0.22 | 8.31E-02 | 2.27E-01 |
| Schaedlerella.glycyrrhizinilytica | 4.05 | 0.20 | 8.35E-02 | 2.28E-01 |
| Ezakiella.coagulans | 4.21 | 1.42 | 8.38E-02 | 2.29E-01 |
| UBA5884.sp900551505 | 4.24 | 0.40 | 8.39E-02 | 2.29E-01 |
| Enterocloster.sp005845215 | 4.25 | 0.28 | 8.43E-02 | 2.29E-01 |
| Phocaeicola.coprocola | 4.31 | 0.27 | 8.43E-02 | 2.29E-01 |
| UBA7597.sp900542935 | 4.34 | 0.34 | 8.43E-02 | 2.29E-01 |
| UBA4871.sp900554535 | 4.37 | 0.07 | 8.46E-02 | 2.30E-01 |
| Anaerotruncus.colihominis | 4.41 | 0.10 | 8.47E-02 | 2.30E-01 |
| Enterobacter.cloacae | 4.51 | 0.68 | 8.68E-02 | 2.35E-01 |
| Eubacterium_G.sp900550135 | 4.53 | 0.12 | 8.67E-02 | 2.35E-01 |
| Odoribacter.sp900548135 | 4.55 | 0.07 | 8.68E-02 | 2.35E-01 |
| CAG.115.sp002492075 | 4.55 | 0.10 | 8.69E-02 | 2.35E-01 |
| QAMX01.sp004555605 | 4.60 | 0.18 | 8.69E-02 | 2.35E-01 |
| HGM11525.sp900770405 | 4.64 | 0.27 | 8.72E-02 | 2.36E-01 |
| Lactonifactor.sp009677585 | 4.71 | 0.19 | 8.74E-02 | 2.36E-01 |
| Clostridium.sp000435835 | 4.72 | 0.23 | 8.77E-02 | 2.37E-01 |
| Blautia_A.sp900551465 | 4.79 | 0.14 | 8.80E-02 | 2.37E-01 |
| Streptococcus.salivarius | 4.84 | 0.23 | 8.85E-02 | 2.39E-01 |
| Merdimonas.sp900553355 | 4.94 | 0.07 | 8.87E-02 | 2.39E-01 |
| CAG.448.sp000433415 | 4.96 | 0.34 | 8.92E-02 | 2.40E-01 |
| UMGS882.sp900757905 | 5.01 | 0.25 | 8.95E-02 | 2.41E-01 |
| Collinsella.sp900556205 | 5.40 | 0.11 | 9.02E-02 | 2.42E-01 |
| Butyricimonas.virosa | 5.45 | 0.20 | 9.03E-02 | 2.42E-01 |
| Clostridium_AQ.sp003481775 | 5.47 | 0.15 | 9.04E-02 | 2.43E-01 |
| CAG.180.sp004556705 | 5.56 | 0.12 | 9.07E-02 | 2.43E-01 |
| CAG.313.sp900539265 | 5.70 | 0.21 | 9.13E-02 | 2.45E-01 |
| NSJ.61.sp003433845 | 5.72 | 0.12 | 9.13E-02 | 2.45E-01 |
| Treponema_D.sp002449305 | 5.74 | 0.05 | 9.16E-02 | 2.45E-01 |
| UBA4871.sp014467055 | 5.88 | 0.05 | 9.17E-02 | 2.45E-01 |
| UBA5905.sp900763035 | 6.38 | 0.56 | 9.17E-02 | 2.45E-01 |
| SFEL01.sp004557245 | 6.79 | 0.23 | 9.18E-02 | 2.46E-01 |
| Alistipes_A.ihumii | 7.23 | 0.24 | 9.23E-02 | 2.46E-01 |
| Alistipes.sp900546065 | 7.53 | 0.26 | 9.26E-02 | 2.47E-01 |
| Lawsonibacter.sp900066825 | 9.25 | 0.10 | 9.27E-02 | 2.47E-01 |
| CAG.475.sp900548005 | 12.26 | 0.14 | 9.36E-02 | 2.49E-01 |

**Supplementary Table 13.** Bacterial metabolic pathways showing different representations between IgA nephropathy nonprogressors and progressors.

| **Feature** | **coef** | **stderr** | **pval** | **qval** |
| --- | --- | --- | --- | --- |
| PWY.5497..purine.nucleobases.degradation.II..anaerobic. | -4.64 | 0.09 | 4.16E-02 | 2.48E-01 |
| GALACT.GLUCUROCAT.PWY..superpathway.of.hexuronide.and.hexuronate.degradation | -2.9 | 0.08 | 4.04E-02 | 2.42E-01 |
| PWY.7807..glyphosate.degradation.III | -2.78 | 0.05 | 4.00E-02 | 2.41E-01 |
| PWY.7286..7..3.amino.3.carboxypropyl..wyosine.biosynthesis | -2.56 | 0.42 | 3.94E-02 | 2.39E-01 |
| PWY.6470..peptidoglycan.biosynthesis.V...beta..lactam.resistance. | -1.83 | 0.05 | 3.90E-02 | 2.37E-01 |
| PWY.6328..L.lysine.degradation.X | -1.46 | 0.7 | 3.62E-02 | 2.26E-01 |
| PYRIDNUCSYN.PWY..NAD.de.novo.biosynthesis.I..from.aspartate. | -1.14 | 0.02 | 3.45E-02 | 2.17E-01 |
| PWY.7199..pyrimidine.deoxyribonucleosides.salvage | -1 | 0.02 | 2.89E-02 | 1.88E-01 |
| PWY.7805...aminomethyl.phosphonate.degradation | -0.99 | 0.06 | 2.80E-02 | 1.83E-01 |
| PWY.6270..isoprene.biosynthesis.I | -0.97 | 0.02 | 2.69E-02 | 1.79E-01 |
| PWY.8125..mevalonate.pathway.IV..archaea. | -0.94 | 0.07 | 2.63E-02 | 1.77E-01 |
| PWY.6174..mevalonate.pathway.II..haloarchaea. | -0.93 | 0.07 | 2.63E-02 | 1.77E-01 |
| PWY.6518..bile.acids.epimerization | -0.9 | 0.05 | 2.43E-02 | 1.68E-01 |
| RHAMCAT.PWY..L.rhamnose.degradation.I | -0.87 | 0.03 | 1.98E-02 | 1.41E-01 |
| NAD.BIOSYNTHESIS.II..NAD.salvage.pathway.III..to.nicotinamide.riboside. | -0.86 | 0.24 | 1.84E-02 | 1.33E-01 |
| THISYNARA.PWY..superpathway.of.thiamine.diphosphate.biosynthesis.III..eukaryotes. | -0.83 | 0.03 | 1.63E-02 | 1.21E-01 |
| AEROBACTINSYN.PWY..aerobactin.biosynthesis | -0.76 | 0.06 | 1.51E-02 | 1.16E-01 |
| PWY.1861..formaldehyde.assimilation.II..assimilatory.RuMP.Cycle. | -0.75 | 0.08 | 1.45E-02 | 1.11E-01 |
| PWY0.162..superpathway.of.pyrimidine.ribonucleotides.de.novo.biosynthesis | -0.72 | 0.04 | 9.10E-03 | 7.52E-02 |
| PWY490.3..nitrate.reduction.VI..assimilatory. | -0.69 | 0.03 | 8.11E-03 | 7.01E-02 |
| RUMP.PWY..formaldehyde.oxidation.I | -0.67 | 0.14 | 6.90E-03 | 6.34E-02 |
| PWY.6953..dTDP.3.acetamido..alpha..D.fucose.biosynthesis | -0.67 | 0.04 | 7.37E-03 | 6.62E-02 |
| PWY.7328..superpathway.of.UDP.glucose.derived.O.antigen.building.blocks.biosynthesis | -0.67 | 0.04 | 7.78E-03 | 6.86E-02 |
| ORNARGDEG.PWY..superpathway.of.L.arginine.and.L.ornithine.degradation | -0.66 | 0.03 | 6.57E-03 | 6.08E-02 |
| ARGDEG.PWY..superpathway.of.L.arginine..putrescine..and.4.aminobutanoate.degradation | -0.58 | 0.03 | 6.57E-03 | 6.08E-02 |
| PWY.7616..methanol.oxidation.to.carbon.dioxide | -0.57 | 0.08 | 4.42E-03 | 4.18E-02 |
| PWY.5855..ubiquinol.7.biosynthesis..early.decarboxylation. | -0.54 | 0.06 | 1.44E-03 | 1.53E-02 |
| PWY0.1533..methylphosphonate.degradation.I | -0.54 | 0.06 | 2.79E-03 | 2.85E-02 |
| PWY.6562..norspermidine.biosynthesis | -0.53 | 0.72 | 3.59E-04 | 4.07E-03 |
| PWY.6628..superpathway.of.L.phenylalanine.biosynthesis | 0.5 | 0.07 | 3.29E-04 | 3.76E-03 |
| PWY.6478..GDP.D.glycero..alpha..D.manno.heptose.biosynthesis | 0.5 | 0.1 | 7.67E-04 | 8.47E-03 |
| PWY.7094..fatty.acid.salvage | 0.51 | 0.07 | 3.10E-04 | 3.58E-03 |
| PWY.5910..superpathway.of.geranylgeranyldiphosphate.biosynthesis.I..via.mevalonate. | 0.52 | 0.05 | 1.57E-04 | 1.86E-03 |
| PWY.7688..dTDP..alpha..D.ravidosamine.and.dTDP.4.acetyl..alpha..D.ravidosamine.biosynthesis | 0.53 | 0.05 | 1.86E-05 | 2.30E-04 |
| PWY.6953..dTDP.3.acetamido..alpha..D.fucose.biosynthesis | 0.54 | 0.08 | 6.19E-06 | 8.05E-05 |
| PWY.8125..mevalonate.pathway.IV..archaea. | 0.55 | 0.15 | 4.34E-06 | 5.70E-05 |
| PWY.5415..catechol.degradation.I..meta.cleavage.pathway. | 0.57 | 0.17 | 6.50E-07 | 9.09E-06 |
| PWY.6174..mevalonate.pathway.II..haloarchaea. | 0.57 | 0.15 | 4.34E-06 | 5.70E-05 |
| PWY.6518..bile.acids.epimerization | 0.6 | 0.11 | 3.76E-08 | 5.43E-07 |
| PWY.5464..superpathway.of.cytosolic.glycolysis..plants...pyruvate.dehydrogenase.and.TCA.cycle | 0.63 | 0.12 | 9.23E-09 | 1.38E-07 |
| P241.PWY..coenzyme.B.biosynthesis | 0.63 | 0.12 | 2.40E-08 | 3.55E-07 |
| AEROBACTINSYN.PWY..aerobactin.biosynthesis | 0.63 | 0.1 | 2.71E-08 | 3.96E-07 |
| PWY.7391..isoprene.biosynthesis.II..engineered. | 0.64 | 0.17 | 6.70E-09 | 1.02E-07 |
| PWY.5677..succinate.fermentation.to.butanoate | 0.65 | 0.09 | 1.12E-09 | 1.75E-08 |
| PWY66.367..ketogenesis | 0.65 | 0.09 | 3.35E-09 | 5.19E-08 |
| PWY.5994..palmitate.biosynthesis..type.I.fatty.acid.synthase. | 0.67 | 0.03 | 9.58E-10 | 1.52E-08 |
| PWY.6167..flavin.biosynthesis.II..archaea. | 0.74 | 0.02 | 6.07E-11 | 9.84E-10 |
| PWY.7332..superpathway.of.UDP.N.acetylglucosamine.derived.O.antigen.building.blocks.biosynthesis | 0.75 | 0.11 | 4.86E-11 | 7.98E-10 |
| PWY.5392..reductive.TCA.cycle.II | 0.76 | 0.05 | 2.24E-11 | 3.78E-10 |
| PWY.6471..peptidoglycan.biosynthesis.IV..Enterococcus.faecium. | 0.77 | 0.07 | 5.65E-15 | 1.00E-13 |
| PWY490.3..nitrate.reduction.VI..assimilatory. | 0.77 | 0.06 | 4.56E-13 | 7.89E-12 |
| PWY0.1533..methylphosphonate.degradation.I | 0.78 | 0.12 | 1.20E-17 | 2.21E-16 |
| THREOCAT.PWY..superpathway.of.L.threonine.metabolism | 0.78 | 0.05 | 2.32E-17 | 4.24E-16 |
| PWY.6920..6.gingerol.analog.biosynthesis..engineered. | 0.79 | 0.12 | 2.76E-20 | 5.34E-19 |
| PWY.6415..L.ascorbate.biosynthesis.V..euglena..D.galacturonate.pathway. | 0.8 | 0.05 | 1.31E-22 | 2.69E-21 |
| PWY.5855..ubiquinol.7.biosynthesis..early.decarboxylation. | 0.81 | 0.1 | 2.87E-26 | 6.29E-25 |
| CATECHOL.ORTHO.CLEAVAGE.PWY..catechol.degradation.to..beta..ketoadipate | 0.84 | 0.14 | 6.47E-37 | 1.85E-35 |
| CRNFORCAT.PWY..creatinine.degradation.I | 0.84 | 0.08 | 7.89E-31 | 1.96E-29 |
| PWY.7807..glyphosate.degradation.III | 0.89 | 0.11 | 3.79E-42 | 1.18E-40 |
| PWY.6165..chorismate.biosynthesis.II..archaea. | 1.01 | 0.02 | 1.04E-42 | 3.34E-41 |
| PWY.6185..4.methylcatechol.degradation..ortho.cleavage. | 1.07 | 0.2 | 2.59E-43 | 8.52E-42 |
| PWY.7312..dTDP..beta..D.fucofuranose.biosynthesis | 1.08 | 0.04 | 6.58E-64 | 2.70E-62 |
| PWY.7805...aminomethyl.phosphonate.degradation | 1.08 | 0.16 | 6.26E-50 | 2.22E-48 |
| PWY.7200..superpathway.of.pyrimidine.deoxyribonucleoside.salvage | 1.08 | 0.06 | 1.40E-45 | 4.85E-44 |
| PWY.6168..flavin.biosynthesis.III..fungi. | 1.15 | 0.01 | 7.13E-74 | 3.47E-72 |
| PWY.7992..superpathway.of.menaquinol.8.biosynthesis.III | 1.41 | 0.02 | 2.43E-75 | 1.23E-73 |
| PWY.6435..4.hydroxybenzoate.biosynthesis.III..plants. | 1.47 | 0.05 | 5.72E-89 | 3.27E-87 |
| PWY.6285..superpathway.of.fatty.acids.biosynthesis..E..coli. | 1.51 | 0.02 | 6.92E-114 | 4.55E-112 |
| GLUCOSE1PMETAB.PWY..glucose.and.glucose.1.phosphate.degradation | 1.53 | 0 | 2.26E-154 | 1.98E-152 |
| PWY.7196..superpathway.of.pyrimidine.ribonucleosides.salvage | 1.97 | 0.02 | 0.00E+00 | 0.00E+00 |
| P108.PWY..pyruvate.fermentation.to.propanoate.I | 2.38 | 0.03 | 0.00E+00 | 0.00E+00 |

**Supplementary Table 14.** Contributing factors determining the observed variation in the gut microbiome-derived metabolic pathways among IgA nephropathy patients only.

| **Variable** | **Df** | **Sum Of Sqs** | **R2** | **F** | **Pr (>F)** |
| --- | --- | --- | --- | --- | --- |
| Gd-IgA1 | 1 | 0.03 | 0.06 | 2.80 | 1.81E-02 |
| BMI | 1 | 0.02 | 0.04 | 1.83 | 8.74E-02 |
| eGFR | 1 | 0.01 | 0.03 | 1.56 | 1.35E-01 |
| Indoxylsulfate | 1 | 0.01 | 0.03 | 1.36 | 2.02E-01 |
| Hematuria | 1 | 0.01 | 0.02 | 1.10 | 3.22E-01 |
| Sex | 1 | 0.01 | 0.02 | 0.73 | 6.25E-01 |
| Progression | 1 | 0.01 | 0.02 | 0.73 | 6.30E-01 |
| LPS | 1 | 0.01 | 0.01 | 0.71 | 6.31E-01 |
| Proteinuria range | 1 | 0.01 | 0.01 | 0.58 | 7.69E-01 |
| Prognosis | 1 | 0.00 | 0.01 | 0.42 | 9.25E-01 |
| Residual | 35 | 0.33 | 0.73 | NA | NA |
| Total | 45 | 0.46 | 1.00 | NA | NA |

**Supplementary Table 15.** Bacterial metabolic pathways showing association with serum Gd-IgA1 levels in IgAN cases only.

| **feature** | **coef** | **stderr** | **pval** | **qval** |
| --- | --- | --- | --- | --- |
| PWY.7332..superpathway.of.UDP.N.acetylglucosamine.derived.O.antigen.building.blocks.biosynthesis | -0.69 | 0.06 | 1.78E-30 | 4.33E-29 |
| CRNFORCAT.PWY..creatinine.degradation.I | -0.67 | 0.04 | 8.29E-56 | 3.03E-54 |
| PWY.6953..dTDP.3.acetamido..alpha..D.fucose.biosynthesis | -0.54 | 0.04 | 4.46E-37 | 1.30E-35 |
| PWY.6435..4.hydroxybenzoate.biosynthesis.III..plants. | -0.41 | 0.02 | 8.74E-70 | 3.70E-68 |
| PWY.6165..chorismate.biosynthesis.II..archaea. | -0.38 | 0.01 | 0.00E+00 | 0.00E+00 |
| PWY.7688..dTDP..alpha..D.ravidosamine.and.dTDP.4.acetyl..alpha..D.ravidosamine.biosynthesis | -0.32 | 0.02 | 1.11E-39 | 3.31E-38 |
| PWY.6174..mevalonate.pathway.II..haloarchaea. | -0.25 | 0.07 | 6.60E-04 | 7.35E-03 |
| PWY.8125..mevalonate.pathway.IV..archaea. | -0.25 | 0.07 | 6.60E-04 | 7.35E-03 |
| PWY.5910..superpathway.of.geranylgeranyldiphosphate.biosynthesis.I..via.mevalonate. | -0.24 | 0.02 | 1.06E-24 | 2.28E-23 |
| PWY.6518..bile.acids.epimerization | -0.24 | 0.05 | 1.34E-05 | 1.73E-04 |
| PWY.6920..6.gingerol.analog.biosynthesis..engineered. | -0.22 | 0.06 | 8.62E-05 | 1.05E-03 |
| PWY.7196..superpathway.of.pyrimidine.ribonucleosides.salvage | -0.22 | 0.01 | 5.49E-141 | 4.24E-139 |
| PWY.7391..isoprene.biosynthesis.II..engineered. | -0.21 | 0.08 | 7.83E-03 | 6.86E-02 |
| PWY.7356..thiamine.diphosphate.salvage.IV..yeast. | -0.15 | 0.06 | 1.90E-02 | 1.36E-01 |
| PWY.7234..inosine.5..phosphate.biosynthesis.III | -0.14 | 0.06 | 2.40E-02 | 1.68E-01 |
| PWY.5180..toluene.degradation.I..aerobic...via.o.cresol. | -0.12 | 0.03 | 8.87E-05 | 1.07E-03 |
| PWY.5484..glycolysis.II..from.fructose.6.phosphate. | -0.11 | 0.05 | 1.55E-02 | 1.17E-01 |
| PWY.7200..superpathway.of.pyrimidine.deoxyribonucleoside.salvage | -0.11 | 0.03 | 2.35E-04 | 2.75E-03 |
| UDPNAGSYN.PWY..UDP.N.acetyl.D.glucosamine.biosynthesis.I | -0.11 | 0.04 | 5.04E-03 | 4.73E-02 |
| PWY.7312..dTDP..beta..D.fucofuranose.biosynthesis | -0.11 | 0.02 | 8.91E-07 | 1.23E-05 |
| GLYCOLYSIS..glycolysis.I..from.glucose.6.phosphate. | -0.10 | 0.05 | 2.80E-02 | 1.83E-01 |
| PWY.6628..superpathway.of.L.phenylalanine.biosynthesis | -0.10 | 0.03 | 2.85E-03 | 2.86E-02 |
| ANAEROFRUCAT.PWY..homolactic.fermentation | -0.09 | 0.04 | 2.00E-02 | 1.42E-01 |
| PWY.6167..flavin.biosynthesis.II..archaea. | -0.08 | 0.01 | 4.80E-11 | 7.98E-10 |
| OANTIGEN.PWY..O.antigen.building.blocks.biosynthesis..E..coli. | -0.07 | 0.03 | 7.41E-03 | 6.62E-02 |
| PWY.6124..inosine.5..phosphate.biosynthesis.II | -0.05 | 0.02 | 3.67E-03 | 3.57E-02 |
| THRESYN.PWY..superpathway.of.L.threonine.biosynthesis | -0.05 | 0.02 | 9.65E-04 | 1.06E-02 |
| PWY.5994..palmitate.biosynthesis..type.I.fatty.acid.synthase. | -0.05 | 0.02 | 2.84E-03 | 2.86E-02 |
| PWY.6123..inosine.5..phosphate.biosynthesis.I | -0.05 | 0.02 | 3.63E-03 | 3.56E-02 |
| SER.GLYSYN.PWY..superpathway.of.L.serine.and.glycine.biosynthesis.I | -0.04 | 0.02 | 3.22E-02 | 2.05E-01 |
| PWY.724..superpathway.of.L.lysine..L.threonine.and.L.methionine.biosynthesis.II | -0.03 | 0.01 | 4.10E-03 | 3.90E-02 |
| GLUCOSE1PMETAB.PWY..glucose.and.glucose.1.phosphate.degradation | 0.03 | 0.00 | 4.61E-34 | 1.24E-32 |
| PWY490.3..nitrate.reduction.VI..assimilatory. | 0.07 | 0.03 | 2.96E-02 | 1.91E-01 |
| PROPFERM.PWY..superpathway.of.L.alanine.fermentation..Stickland.reaction. | 0.08 | 0.02 | 1.61E-05 | 2.01E-04 |
| PWY.8188..L.alanine.degradation.VI..reductive.Stickland.reaction. | 0.08 | 0.02 | 1.61E-05 | 2.01E-04 |
| PWY.8189..L.alanine.degradation.V..oxidative.Stickland.reaction. | 0.08 | 0.02 | 1.61E-05 | 2.01E-04 |
| PWY.6507..4.deoxy.L.threo.hex.4.enopyranuronate.degradation | 0.11 | 0.06 | 3.80E-02 | 2.35E-01 |
| ARG.POLYAMINE.SYN..superpathway.of.arginine.and.polyamine.biosynthesis | 0.12 | 0.05 | 1.38E-02 | 1.08E-01 |
| POLYAMSYN.PWY..superpathway.of.polyamine.biosynthesis.I | 0.13 | 0.06 | 2.62E-02 | 1.77E-01 |
| PWY.5677..succinate.fermentation.to.butanoate | 0.13 | 0.04 | 1.81E-03 | 1.92E-02 |
| PWY.5494..pyruvate.fermentation.to.propanoate.II..acrylate.pathway. | 0.13 | 0.02 | 1.23E-07 | 1.76E-06 |
| PWY.7013...S..propane.1.2.diol.degradation | 0.20 | 0.08 | 1.05E-02 | 8.63E-02 |
| PWY.6168..flavin.biosynthesis.III..fungi. | 0.20 | 0.00 | 0.00E+00 | 0.00E+00 |
| PWY0.1297..superpathway.of.purine.deoxyribonucleosides.degradation | 0.21 | 0.09 | 1.68E-02 | 1.24E-01 |
| PWY66.367..ketogenesis | 0.23 | 0.04 | 1.98E-07 | 2.79E-06 |
| PWY.7315..dTDP.N.acetylthomosamine.biosynthesis | 0.30 | 0.14 | 3.85E-02 | 2.35E-01 |
| HEME.BIOSYNTHESIS.II..heme.b.biosynthesis.I..aerobic. | 0.31 | 0.13 | 1.65E-02 | 1.22E-01 |
| PWY.6143..CMP.pseudaminate.biosynthesis | 0.32 | 0.02 | 8.61E-44 | 2.90E-42 |
| PWY0.781..aspartate.superpathway | 0.34 | 0.11 | 2.70E-03 | 2.78E-02 |
| P4.PWY..superpathway.of.L.lysine..L.threonine.and.L.methionine.biosynthesis.I | 0.34 | 0.11 | 2.65E-03 | 2.74E-02 |
| PWY.5347..superpathway.of.L.methionine.biosynthesis..transsulfuration. | 0.35 | 0.14 | 1.30E-02 | 1.03E-01 |
| MET.SAM.PWY..superpathway.of.S.adenosyl.L.methionine.biosynthesis | 0.35 | 0.14 | 1.31E-02 | 1.03E-01 |
| METSYN.PWY..superpathway.of.L.homoserine.and.L.methionine.biosynthesis | 0.36 | 0.14 | 1.30E-02 | 1.03E-01 |
| PWY.5415..catechol.degradation.I..meta.cleavage.pathway. | 0.37 | 0.08 | 2.68E-06 | 3.67E-05 |
| HOMOSER.METSYN.PWY..L.methionine.biosynthesis.I | 0.37 | 0.15 | 1.23E-02 | 9.89E-02 |
| PWY.7159..3.8.divinyl.chlorophyllide.a.biosynthesis.III..aerobic..light.independent. | 0.39 | 0.08 | 2.73E-06 | 3.70E-05 |
| CHLOROPHYLL.SYN..3.8.divinyl.chlorophyllide.a.biosynthesis.I..aerobic..light.dependent. | 0.40 | 0.09 | 3.52E-06 | 4.72E-05 |
| PWY.6478..GDP.D.glycero..alpha..D.manno.heptose.biosynthesis | 0.41 | 0.05 | 9.99E-18 | 1.90E-16 |
| PWY.6396..superpathway.of.2.3.butanediol.biosynthesis | 0.46 | 0.06 | 2.79E-16 | 5.02E-15 |
| PWY0.1533..methylphosphonate.degradation.I | 0.50 | 0.05 | 6.15E-21 | 1.23E-19 |
| P241.PWY..coenzyme.B.biosynthesis | 0.50 | 0.05 | 8.01E-21 | 1.57E-19 |
| GLUCARDEG.PWY..D.glucarate.degradation.I | 0.51 | 0.21 | 1.62E-02 | 1.21E-01 |
| P108.PWY..pyruvate.fermentation.to.propanoate.I | 0.54 | 0.01 | 0.00E+00 | 0.00E+00 |
| PWY.7807..glyphosate.degradation.III | 0.57 | 0.04 | 1.35E-40 | 4.13E-39 |
| PWY1G.0..mycothiol.biosynthesis | 0.60 | 0.06 | 8.22E-23 | 1.71E-21 |
| PWY.7094..fatty.acid.salvage | 0.63 | 0.04 | 2.82E-72 | 1.28E-70 |
| PWY.6285..superpathway.of.fatty.acids.biosynthesis..E..coli. | 0.63 | 0.01 | 0.00E+00 | 0.00E+00 |
| GALACTARDEG.PWY..D.galactarate.degradation.I | 0.65 | 0.22 | 3.45E-03 | 3.41E-02 |
| GLUCARGALACTSUPER.PWY..superpathway.of.D.glucarate.and.D.galactarate.degradation | 0.65 | 0.22 | 3.45E-03 | 3.41E-02 |
| PWY0.1337..oleate..beta..oxidation | 0.67 | 0.03 | 7.01E-85 | 3.69E-83 |
| HEME.BIOSYNTHESIS.II.1..heme.b.biosynthesis.V..aerobic. | 0.74 | 0.33 | 2.67E-02 | 1.78E-01 |
| PWY.5723..Rubisco.shunt | 0.75 | 0.36 | 3.83E-02 | 2.35E-01 |
| PWY.5392..reductive.TCA.cycle.II | 0.76 | 0.03 | 8.92E-186 | 1.07E-183 |
| PWY.7616..methanol.oxidation.to.carbon.dioxide | 0.77 | 0.07 | 4.14E-27 | 9.54E-26 |
| PROTOCATECHUATE.ORTHO.CLEAVAGE.PWY..protocatechuate.degradation.II..ortho.cleavage.pathway. | 0.77 | 0.36 | 3.20E-02 | 2.05E-01 |
| ARGDEG.PWY..superpathway.of.L.arginine..putrescine..and.4.aminobutanoate.degradation | 0.78 | 0.03 | 3.23E-161 | 3.04E-159 |
| ORNARGDEG.PWY..superpathway.of.L.arginine.and.L.ornithine.degradation | 0.78 | 0.03 | 3.23E-161 | 3.04E-159 |
| PWY.7805...aminomethyl.phosphonate.degradation | 0.79 | 0.05 | 1.73E-57 | 6.67E-56 |
| PWY.5464..superpathway.of.cytosolic.glycolysis..plants...pyruvate.dehydrogenase.and.TCA.cycle | 0.80 | 0.05 | 2.05E-56 | 7.68E-55 |
| PWY.7446..sulfoquinovose.degradation.I | 0.81 | 0.05 | 1.05E-70 | 4.59E-69 |
| THREOCAT.PWY..superpathway.of.L.threonine.metabolism | 0.84 | 0.02 | 8.71E-285 | 1.43E-282 |
| AEROBACTINSYN.PWY..aerobactin.biosynthesis | 0.89 | 0.05 | 4.73E-73 | 2.22E-71 |
| PWY.5855..ubiquinol.7.biosynthesis..early.decarboxylation. | 1.08 | 0.04 | 2.72E-128 | 1.98E-126 |
| GLYCOLYSIS.TCA.GLYOX.BYPASS..superpathway.of.glycolysis..pyruvate.dehydrogenase..TCA..and.glyoxylate.bypass | 1.08 | 0.03 | 1.01E-236 | 1.32E-234 |
| PWY.6562..norspermidine.biosynthesis | 1.41 | 0.53 | 7.64E-03 | 6.79E-02 |
| CATECHOL.ORTHO.CLEAVAGE.PWY..catechol.degradation.to..beta..ketoadipate | 1.51 | 0.07 | 8.25E-94 | 5.16E-92 |
| PWY.6185..4.methylcatechol.degradation..ortho.cleavage. | 1.53 | 0.08 | 6.04E-85 | 3.31E-83 |
| PWY.7409..phospholipid.remodeling..phosphatidylethanolamine..yeast. | 1.97 | 0.81 | 1.54E-02 | 1.17E-01 |
